# VIII International Symposium on Thysanoptera and Tospoviruses September 11–15, 2005 Asilomar, Pacific Grove, California

**DOI:** 10.1673/031.007.2801

**Published:** 2007-05-04

**Authors:** Organized by:, Diane Ullman, James Moyer, Rob Goldbach, Gerald Moritz

**Affiliations:** ^1^ Department of Entomology, University of California-Davis; ^2^ Department of Plant Pathology, North Carolina State University; ^3^ Laboratory of Virology, Wageningen Agricultural University; ^4^ Developmental Biology, Martin-Luther-University Halle-Wittenberg

Abstracts are listed in alphabetical order by the last name of the senior author.

