# Supplementary material for: VIII International Symposium on Thysanoptera and Tospoviruses September 11–15, 2005 Asilomar, Pacific Grove, California
Source: J Insect Sci. 2007 May 4;7:28. doi: 10.1673/031.007.2801 (PMC2999430; doi:10.1673/031.007.2801)
Supplement: Related texts

# Research article: Diversity of *Tomato spotted wilt virus* isolates in the field

**DOI:** 10.1673/031.007.2802

**Published:** 2007-05-04

**Authors:** S Adkins, DJ Lewandowski

**Affiliations:** ^1^ USDA-ARS, U.S. Horticultural Research Laboratory, 2001 South Rock Road, Fort Pierce, FL 34945, USA; ^2^ Department of Plant Pathology, University of Florida, Citrus Research and Education Center, Lake Alfred, FL 33850, USA. sadkins@ushrl.ars.usda.gov

*Tomato spotted wilt virus* (TSWV) infects tomatoes and a wide range of other crops across temperate, subtropical and tropical regions throughout the world. A broad array of TSWV symptoms is observed on crops in Florida. In tomato foliage, for instance, necrotic ring and line patterns, necrotic spots, leaf rolling and leaf bronzing/purpling are common. This variability in symptoms may reflect diversity in the virus population. A study of the TSWV population in Florida was initiated in an effort to examine its diversity. A better understanding of the TSWV population structure will aid development of rational disease management strategies.

# Research article: Flower thrips, oviposition and dispersion behavior in early-season blueberries

**DOI:** 10.1673/031.007.2803

**Published:** 2007-05-04

**Authors:** HA Arévalo, OE Liburd

**Affiliations:** Entomology and Nematology Department, University of Florida, Gainesville, FL 32611 USA. aleareva@ufl.edu

United States is the largest producer of blueberries with an approximate value of 137,000 tons/year. This represents 65% of the total production worldwide. Early-season blueberries produced only in Florida and southern Georgia between the months of April and May have prices that can be three to five times higher than regular-season blueberries in northern US states. Flower-thrips from the genus *Frankliniella* are considered as key pests for early-season blueberries in the southeastern United States. Based on observations conducted between 2003 and 2005 during the blueberry flowering season, we determined that the predominant flower-thrips species of blueberries in Florida is *F. bispinosa* (Morgan), and in southeastern Georgia is *F. tritici* (Fitch). Other species present include *F. occidentalis* (Pergande), *F. fusca* (Hinds), *Thrips hawaiiensis* (Morgan), and *T. pini* Uzel. A series of observations in oviposition behavior and populations dynamics were made in Southern highbush (*Vaccinium corymbosum* × *V. darrowi*) and Rabbiteye blueberry (*V. ashei*), the two main species grown in Florida and Southern Georgia. The main objective of this work was to understand the oviposition preferences, movement and distribution of flower-thrips in early-season blueberries, with the ultimate goal of establishing an IPM program for controlling thrips. The results of our observations show a significantly higher number of thrips emerging from flower petals than from ovaries, styles or fruits (*F* = 19.16; *df* = 3, 158; *P* < 0.0001) of flowers collected in the field. This indicates a preference to oviposit on the petals than in any other tissues of the blueberry flower. There were no significant differences in thrips emergence between the two blueberry species evaluated, Rabbiteye (3.11 ± 1.2) and Southern Highbush (4.0 ± 1.5) (*F* = 0.01, *df* = 1,38; *P* = 0.93). At the beginning of the season when the flowers begin to open, thrips infestation is very sporadic and populations are relatively low. Flower-thrips clusters or “hot-spots” are defined 7 days after flowers begin to open. Thrips gradually increase in area and population for approximately 2 weeks. Thrips populations in these “hot spots” increase exponentially for approximately 14 days, until 90% of the flowers are open, then it declines until the thrips virtually disappear at petal fall. At this time only fruits are standing in the field illustrating a correlation with the phenology of the flowers. Based on this time-line and observations conducted in the laboratory, thrips may have at least one generation on blueberry flowers, affecting fruit quality by leaving scars from feeding, and injuring reproductive tissues by ovipositing, and emerging from the ovary. Thrips also affect and quantity of blueberries by dehydrating flowers that have been severely attacked.

# Research article: Natural occurrence and genetic diversity of *Tospovirus* isolates from India

**DOI:** 10.1673/031.007.2804

**Published:** 2007-05-04

**Authors:** M Bhanupriya, P Srinivasulu, UB Zehr, KS Ravi

**Affiliations:** ^1^ Mahyco Research Center, Jalna-Aurangabad Road, Dawalwadi, Jalna-431203, Maharashtra, India.; ^2^ Department of Virology, S.V.University, Tirupati-517502, Andhra Pradesh, India Ravi.Kankanallu@mahyco.com

*Tospoviruses* are one of the major constraints in vegetable production next to geminiviruses in India. The spotted wilt in tomato, caused by peanut bud necrosis virus (PBNV) which is widely prevalent and it is one of the significant yield-limiting factors. Surveys were conducted during 2002–05 in the potential vegetable growing regions of Maharashtra, Andhra Pradesh, Karnataka and Tamil Nadu for the detection of tospoviruses and their natural occurrence. The incidence of spotted wilt in tomato varied from 2 to 87%. Field isolates showing typical spotted wilt and related symptoms was detected by DAC-ELISA using PBNV specific antisera, some of the hosts include tomato, chili, brinjal, peanut, greengram, soybean, blackgram, carrot, field bean and watermelon. In order to establish the genetic diversity among tospovirus isolates, the representative virus isolates were initially purified in cowpea cv. C-152 and characterized the NP gene sequences. Molecular analysis showed that the NP gene sequences from 36 isolates among nine different crops revealed very high degree of homology ie 94–99% at nucleotide and 94.9–100% at amino acid level with the reference Indian PBNV sequence (U27809). The characterized PBNV isolates were also compared with the other tospovirus members which include watermelon bud necrosis virus (WBNV) and watermelon silver mottle virus, which shares 79.8–81.3% and 83.7–87.3% homology at nucleotide & amino acid level respectively. In addition to PBNV, three other distinct tospoviruses viz., WBNV, IYSV and peanut yellow spot virus isolates were encountered in watermelon, onion and peanut crops respectively.

# Research article: Characterization of *Frankliniella fusca* dispersal and *Tomato spotted wilt virus* incidence within fields from wild plant hosts

**DOI:** 10.1673/031.007.2805

**Published:** 2007-05-04

**Authors:** AL Beaudoin, GG Kennedy

**Affiliations:** North Carolina State University, Department of Entomology, Raleigh, NC, USA. alparodi@ncsu.edu

Spatial dispersal patterns of *Frankliniella fasca* (Thripidae), a vector of the tomato spotted wilt virus (TSWV), are not well understood. A study was conducted in three fields of bare soil in North Carolina to determine if the number of adult *F. fusca* caught on yellow, sticky traps exhibited a gradient varying with distance from a natural weedy source containing known hosts of *F. fasca*. Yellow, sticky aerial traps were placed 0.76 meters above the soil in recently disked fields. The traps were placed in a grid and the nearest potential sources of thrips on one or more edges of each field were identified. Traps remained in the field for one week and were collected before any vegetation emerged in the field. The number of *F. fusca* per trap was determined. The data revealed no gradients in numbers of thrips per trap over distances of up to 500 meters from a potential thrips source. In late July, a second study was conducted in three tobacco fields to determine if TSWV incidence exhibited a gradient with changes in distance from potential thrips and TSWV sources. Rows of tobacco plants were visually scored for the presence of TSWV every 15.24 meters, resulting in a grid. The nearest potential sources of thrips and TSWV were identified. No gradient in TSWV incidence was found in distances of up to 200 meters. These studies suggest that management of weed hosts of TSWV around field margins may not be an effective strategy to reduce spread of tomato spotted wilt virus into susceptible crops.

# Research article: Thrips and *Tospoviruses* benefit from jointly challenging plants

**DOI:** 10.1673/031.007.2806

**Published:** 2007-05-04

**Authors:** B Belliure, A Janssen, MW Sabelis

**Affiliations:** ^1^ IBED, Institute for Biodiversity and Ecosystem Dynamics, Section Population Biology. University of Amsterdam. PO Box 94084 1090 GB Amsterdam, The Netherlands; ^2^ Present address: IVIA, Valencian Institute for Agricultural Research, Entomology Unit. Apartado Oficial, 46113 Moncada (Valencia), Spain. belliure@ivia.es

Plants are the target of various attackers, such as herbivorous arthropods and plant pathogens. Constitutive defences to these attacks (spines, hard cuticle, etc.) are costly when attackers are not present. Therefore, many plants possess inducible defences that are switched on only upon attack. Induced responses to herbivory are mediated via several metabolic pathways, in particular the Jasmonic Acid (JA) pathway. Defences induced by herbivores involve changes in the quality of attacked plants that affect the fitness of the herbivore (direct defence), and the emission of plant volatiles that attract natural enemies of the herbivore towards attacked plants (indirect defence). As a result, herbivore-induced plants become hosts of inferior quality for herbivorous arthropods, and are therefore less attractive for conspecific or heterospecific herbivores. Pathogens induce anti-pathogen resistance in plants through the Salicylic Acid (SA) pathway. Anti-herbivore and anti-pathogen defensive pathways cross talk in several plant-pathogen systems: up-regulation of the SA pathway results in down-regulation of the JA pathway. This negative cross talk can be exploited by vectors of pathogens, because induction of the anti-pathogen pathway reduces investment of the plant in anti-herbivore defence. In turn, vector-borne pathogens receive a benefit from this cross talk because a pathogen-infected plant with a reduced anti-herbivore defence represents a good host plant for the herbivorous vector of the pathogen. This is the case for *Tospoviruses* that are vectored by thrips. Thrips induce anti-herbivore defences in the plants that are detrimental for thrips. However, adult thrips are often attracted to tospovirus-infected plants, in spite of these being induced by thrips. Recent research showed that *Frankliniella occidentalis* actually benefited from attking plants infected with Tomato spotted wilt virus; survival and developmental rates of thrips larvae were as high on virus-induced (and thus also herbivore-induced) plants as on clean, uninduced plants, and higher than on plants with thrips damage. This is in agreement with the described JA/SA cross-talk. The interaction between the two pathways has several ecological implications. We show that spider mites, another herbivorous arthropod that do not vector the virus, can benefit from plants being infected with the virus. However, this benefit is not due to the negative effect of the virus on the defensive response of the plants to thrips damage. Furthermore, we show that the shorter developmental rate of thrips larvae that develop on plants with virus results in reduced vulnerability to attacks by at least some of its natural enemies. We propose that mechanisms to reduce direct and indirect plant defences against vectors may have evolved in vector-borne plant pathogens, thereby promoting spread of the pathogen.

# Research article: Use of entomopathogenic nematodes for the management of western flower thrips and tospoviruses

**DOI:** 10.1673/031.007.2807

**Published:** 2007-05-04

**Authors:** J Bennison, K Maulden, M Tomiczek, J Morris, I Barker, N Boonham, N Spence

**Affiliations:** ^1^ ADAS Boxworth, Boxworth, CB23 4NN,UK; ^2^ Central Science Laboratory, Sand Hutton, York, Y041 1LZ, UK. jude.bennison@adas.co.uk

Some of the major UK growers of chrysanthemums and other protected ornamental crops are using entomopathogenic nematodes (epns) for biological control of western flower thrips (WFT), *Frankliniella occidentalis*. Weekly foliar sprays of *Steinernema feltiae* (‘Nemasys F’ ®) are used throughout the life of the crop and these generally give good control of thrips. However, use of epns for thrips control is expensive and this is inhibiting more growers from adopting the technique. In addition, control on chrysanthemums can sometimes be inadequate during the summer when thrips pressure is high. Current Defra-funded research aims to fill gaps in knowledge on how epns control WFT populations and whether this control is sufficient to reduce the spread and severity of *Tomato spotted wilt virus* (TSWV) on chrysanthemums. Results indicate that epns reaching the growing medium during foliar application could play an important role in WFT control by killing the ground-dwelling thrips life stages. This information should enable the development of techniques to make the use of epns more effective and economic. In a glasshouse experiment, weekly foliar applications of ‘Nemasys F’ were made to two sequential crops of pot chrysanthemums, cv. ‘Swing Time’, which had been infested with viruliferous WFT larvae. The nematodes reduced mean numbers of WFT per plant, delayed plant infection with TSWV and reduced the symptom expression of TSWV.

# Research article: Temporal and spatial distribution of thrips in Israel

**DOI:** 10.1673/031.007.2808

**Published:** 2007-05-04

**Authors:** D Ben-Yakir, P Jasrotia, M Chen

**Affiliations:** Entomology Department, Institute of Plant Protection, ARO, The Volcani Center, P.O. Box 6, Bet-Dagan, Israel 50250. benyak@volcani.agri.gov.il

We used pole traps to determine the annual and daily variations in the migration activity of *Thrips tabaci* and *Frankliniella occidentalis*. The pole traps were 3m high and were placed near walk-in tunnels and in open fields at 4 different geographical locations in Israel. Thrips populations were peaked between April and June depending on the environmental conditions at each location. Twice as many thrips were caught on the pole at the eastern entrance of the tunnels than at the western entrance. At almost all sits, the highest numbers of thrips were caught on the eastern side of the poles (40–50%) and the lowest numbers were caught on the western side (10–20%). Annually, about 75% of the thrips were caught before noon time, and about 50% were caught below the height of 70 cm. In preliminary trials in which the eastern entrance of the tunnel was covered, it reduced thrips entry significantly with only a slight effect on ventilation efficacy. This information can be used to reduce the risk of thrips entry into greenhouses. Rabbit immunoglobulin G (R-IgG) was used successfully as an external mark for thrips. Females of both *Thrips tabaci* and *Frankliniella occidentalis* were marked with R-IgG solution (1 mg/ml water with 1% Tween 20) by the contact exposure method. Determining the retention of the mark was done by running the rinsing solutions of individual thrips in an enzyme—linked immunosorbant assay (ELISA). The sandwich ELISA method was used with an additional biotin-avidin step that increased the assay's sensitivity by 3–5 folds. When marked thrips were kept in the laboratory on bean pods they all scored positive 6 days after marking (DAM). All marked thrips scored positive 2 DAM when kept in the laboratory on detached marigold flowers. When marked and unmarked thrips were placed together on these flowers some mark had transferred and 10–20% of the unmarked thrips turned positive. Under field conditions, a gradual decrease in the mean optical density levels of the marked thrips was observed over time. On sticky pole traps, 100%, 85% and 15% of the marked *T. tabaci* scored positive by the 3rd, 6th and 9th DAM, respectively. Under the same conditions 100% and 15% of the marked *F. occidentalis* scored positive by the 6th and 9th DAM, respectively. Similar rates of positive scores were recorded for marked *T. tabaci* kept on chive plants for 6 DAM. The retention of the R-IgG mark decreased significantly under conditions of wetness and high humidity. The suitability of this marking method for dispersal studies of these important pests need to be evaluated now.

# Research article: Spatial dynamics of spotted wilt in georgia grown tobacco and peanut

**DOI:** 10.1673/031.007.2809

**Published:** 2007-05-04

**Authors:** P Bertrand, A Culbreath

**Affiliations:** Department of Plant Pathology, The University of Georgia. bertrand@uga.edu

The spatial development of spotted wilt has been studied in tobacco and peanut. Tobacco data from trials conducted between 1989–2004 comprising ∼2200 individual reps with > 100 plants was subjected to ordinary runs analysis. The results were tabulated by disease incidence classes ranging from <11% – >71% at 10% intervals. In the four disease incidence classes ranging from <11% – 40.9% aggregation occurred in 10–13% of the cases. In the four disease incidence classes from 41% – >71% there was a steadyilar conclusion for both peanut and tobacco. Secondary infection is biologically possible in both crops however data from spatial analysis and other trials suggest that the majority of visible disease results from primary infection.

# Research article: Influence of morphological and chemical constituents of plant foliage on thrips host selection among the botanical collections in the Temperate House, Royal Botanic Gardens, Kew

**DOI:** 10.1673/031.007.2810

**Published:** 2007-05-04

**Authors:** AS Scott Brown, NC Veitch, MJS Simmonds

**Affiliations:** Jodrell Laboratory, Royal Botanic Gardens, Kew, Richmond, Surrey, TW9 3AB, UK. a.scott-brown@rbgkew.org.uk

One of the primary aims of the Royal Botanic Gardens (RBG), Kew, is to conserve its unique living collections of 33,000 plant species, many of which are classified as rare and endangered. Thrips have persisted as a threat to plant species contained in the display glasshouses since the introduction of IPM programs at Kew in the early 1990s. The genetic and phenotypic diversity of plants housed together in artificial conditions has lowered the efficiency of biological control predators which have been used successfully to control invasive thrips species on protected crops in the commercial horticultural sector. We have monitored and documented plants among the collections in the Temperate House, RBG Kew, as hosts or non-hosts to two invasive thrips; *Heliothrips haemorrhoidalis* (Bouché), and *Frankliniella occidentalis* (Pergande) and further investigations have highlighted species of host plants where the effectiveness of the predators; *Neoseiulus cucumeris* (Oudemans), *Iphiseius degenerans* (Berlese) and *Orius laevigatus* (Fieber) were significantly reduced. This study aims to highlight the morphological and chemical differences among the foliage of the hosts and non-hosts of *H. haemorrhoidalis*. Scanning electron microscope techniques were used to describe the morphology of the leaf surface of 19 species in order to help identify which morphological features could play a role in the selection of plant species by thrips from among a diverse botanical collection. The results showed *H. haemorrhoidalis* had a preference for species with leaves that were coriaceous, with one or both surfaces being smooth. Plants evading these thrips commonly possessed glandular trichomes and waxes present on the surfaces of leaves were observed to be of dense, non-uniform forms, thus potentially disrupting feeding and oviposition of *H. haemorrhoidalis*. In addition, detailed analysis of the phytochemical profiles of the leaves of non-hosts are in progress to identify which groups of compounds deter thrips, and which compounds present in hosts may result in a decrease in efficiency of the introduced predators. Initial studies have focused on the profiles of two non-hosts of *H. haemorrhoidalis* and several isolated compounds present in the foliage of these species have shown levels of activity against immature stages of *F. occidentalis* in controlled bioassay tests. Evaluating the roles of morphological and chemical characteristics of non-host plants in plant-thrips-predator interactions can serve to advance our methods in controlling thrips pests in all sectors of Horticulture.

# Research article: “There's no place like home” — *Tospoviruses* in Kansas

**DOI:** 10.1673/031.007.2811

**Published:** 2007-05-04

**Authors:** LR Campbell, C Webb, L Westervelt

**Affiliations:** ^1^ Department of Entomology, Kansas State University, Manhattan KS 66506.; ^2^ USDA, APHIS, PPQ, Department of Plant Pathology, Kansas State University, Manhattan KS 66506.; ^3^ Department of Horticulture, Forestry and Recreation Resources, Kansas State University, Manhattan KS 66506. lcambel@ksu.edu

Kansas State University has an active Horticultural Therapy program and teaching programs in Plant Pathology, Horticulture, Forestry and Master Gardener programs. These courses all contain a hands-on training component, which takes place in the Throckmorton Greenhouse Complex. This results in increased human traffic and plant movement between the greenhouses. These activities increase the chance for thrips and their vectoring of Tomato Spotted Wilt Virus (TSWV) and Impatient Necrotic Spot Virus (INSV). On-going long term experiments, some involving tomatoes and other virus-susceptible plants, are also located in the complex. In addition, the K-State Gardens, located adjacent to the greenhouse complex, are a major tourist attraction and displays many tospovirus-susceptible plant species. Early in 2004, bedding plants in the Throckmorton Greenhouse Complex showed symptoms indicative of tospovirus infections. Initial testing using Immuno Strips (Agdia Corp.) verified TSWV/INSV in 4 of 22 plants. Funding was acquired with the purpose of developing and implementing policy to control tospovirus in the K-State Horticulture, Forestry & Recreation Resources (HFRR) greenhouses. Totally, 677 plants were sampled from 16 greenhouses, with in (16.39%) testing positive. These included 47 species from 29 families, with 23 species not found on other host lists. From this work, greenhouse management policies have been instituted. Weekly monitoring with sticky cards and elimination of infected plant material will control future tospovirus outbreaks.

# Research article: Improved *Tospovirus* management systems for greenhouse flower crops

**DOI:** 10.1673/031.007.2812

**Published:** 2007-05-04

**Authors:** C Casey, S Sutliff-Shipley

**Affiliations:** Department of Entomology, North Carolina State University, Raleigh, NC 27695, USA. chris_casey@ncsu.edu

The state of North Carolina has the fourth largest ornamental horticulture industry in the U.S., valued at about $1billion per year. Thrips and tospoviruses cause significant losses for greenhouse flower growers in North Carolina. In greenhouses the predominant virus is impatiens necrotic spot virus (INSV), although mixed infections with TSWV may be present. The western flower thrips, *Frankliniella occidentalis*, is considered to be the most important vector. A priority for improved greenhouse INSV management is some type of early warning system that would alert growers to the presence of infected thrips before crop symptoms are seen. Indicator plants develop characteristic symptoms within a few days after feeding by viruliferous thrips and serve as an alert to their presence. Since this system will only be effective if the indicator plant is more attractive to the viruliferous thrips than the crops they are intended to protect, we evaluated the relative attractiveness of two indicator plants, petunia and fava bean, and several greenhouse crops, to viruliferous *F. occidentalis*. Differences between indicator plants species in attractiveness were observed, and in some cases the crop plant was more attractive to thrips than the indicator. Since understanding thrips movement is key to understanding how epidemics develop, we also examined how pesticides might stimulate thrips movement and disease spread. If a pesticide is distasteful (i.e. repellent) a short tasting probe may stimulate vector movement without providing a lethal dose. Common greenhouse pesticides used for thrips and weed control were found to be repellent to *F. occidentalis*.

# Research article: Exploration of gut bacteria in *Frankliniella occidentalis* (Pergande), Western Flower Thrips

**DOI:** 10.1673/031.007.2813

**Published:** 2007-05-04

**Authors:** L Chanbusarakum, D Ullman

**Affiliations:** University of California-Davis, Department of Entomology, One Shields Avenue, Davis, CA 95616. ljchan@ucdavis.edu

Symbiotic bacteria often have a complex association with their host, be it human or arthropod. Many insects possess symbionts, though it is often difficult to determine the intricacies of their relationships. In one such case, facultative bacteria have been discovered in the hindgut of a major crop pest and virus vector, the Western flower thrips (WFT), *Frankliniella occidentalis* (Pergande) (Thysanoptera: Thripidae). The bacteria can be cultured outside thrips and are horizontally transmitted among WFT. Several thrips bacterial isolates have been studied in the Netherlands. Molecular data indicated thrips symbionts were similar to *Escherichia coli*, though biochemical properties suggested these microbes might actually be most similar to *Erwinia*. We focused on the bacterial flora of thrips originating from the Hawaiian Islands. Phenotypic data show that bacteria from thrips originating in the Hawaiian Islands and Netherlands were very similar. Molecular phylogeny based on the 16s rRNA gene suggested 2 separate groups of thrips bacteria were present in isolates from both geographic regions. One of the groups fell out closely with some *Erwinia*; however, bootstrap values for this classification were very low. Sound bootstrap values were not found for any of the relationships for the second group. The similarity between the microbes from different thrips in different locations and raised on different plant material suggest that the relationship between the bacteria and WFT are relatively stable and are not incidental infections arising from their environment.

# Research article: The nucleocapsid and the non structural small proteins of *Tomato spotted wilt virus* (TSWV) strain Br01 are not sufficient for eliciting lethal necrosis in *Nicotiana benthamiana* and local necrotic lesions on *Nicotiana tabacum*

**DOI:** 10.1673/031.007.2814

**Published:** 2007-05-04

**Authors:** M Ciuffo, D Pacifico, M Turina

**Affiliations:** Istituto di Virologia Vegetale, CNR, Torino 10135, Italy. m.ciuffo@ivv.cnr.it

We detected a consistent different phenotype on *Nicotiana benthamiana* and *N. tabacum* between the Br01 isolate and the Italian isolate p272. Based on the phenotype of a reassortant strain carrying the S genomic segment of p272, and the M and L genomic segments from strain Br01, we concluded that the small segment of Br01 is necessary for eliciting lethal necrosis on *N. benthamiana* and local necrotic lesions on *N. tabacum*. The aim of this work was to evaluate if the nucleocapsid and non-structural small proteins encoded by the small genomic segment of strain Br01 were able to cause the distinctive symptoms, alone or in combination. The two allelic variants (the Br01 and p272) of each protein were transiently expressed *in planta* through agroinfiltration. Symptoms were monitored in order to detect reaction specific to the Br01 alleles. As a positive control for a viral protein causing lethal necrosis and necrotic local lesion, we infiltrated p19 of *Tomato bushy stunt virus* (TBSV); as negative control, the protein green fluorescent protein was inserted in the pBin61 vector. Accumulation of nucleocapsid and non-structural small proteins were monitored through western blot analysis. Green fluorescent protein accumulation was monitored visually under UV light observation. All the different alleles of the nucleocapsid and NSs proteins readily accumulated in the infiltrated *N. benthamiana* and *N. tabacum* leaves; no necrotic reaction was observed in any of the single or combined agro-infiltration. A necrotic response was observed when p19 protein was infiltrated in both *N. benthamiana* and *N. tabacum*. Our results suggest that non-structural small proteins Br01 and N Br01 cannot cause a necrotic response alone or in combination. Our hypothesis involves the requirement of different regions of the M and L segmentsof the genome.

# Research article: A sequence specific real-time PCR (TaqMan®) assay for *Thrips palmi* (Thysanoptera: Thripidae); Its use and advantages as a molecular quarantine identification tool

**DOI:** 10.1673/031.007.2815

**Published:** 2007-05-04

**Authors:** DW Collins, K Walsh, N Boonham, R Glover, I Barker

**Affiliations:** Central Science Laboratory, Sand Hutton, York YO41 1LZ, United Kingdom. dom.collins@csl.gov.uk

The polyphagous pest *Thrips palmi* Karny has become a species of major quarantine concern, often intercepted on plant material in international trade. The ability to rapidly identify the species is a critical factor that will help determine the success of any campaign to prevent its establishment in Europe. Confirmed morphological identification to species is limited to the adult thrips, yet quarantine diagnostic laboratories frequently have to make identifications with only larvae available, often with perishable commodities at stake. In this study, RAPD analysis was performed to identify 19 putative SCAR markers, which were screened by southern blot analysis; one marker was sequenced and a real-time PCR (TaqMan) assay developed (Walsh K *et al*. 2005. *Journal of Applied Entomology* 129: 272–279). The assay was screened against 21 thrips species including 10 other species of the genus *Thrips* and found to be specific to *T. palmi*. The speed, specificity, simplicity and robustness of the assay are all reasons why a real-time PCR format was selected for specific use as a quarantine entomology diagnostic tool in preference to other available molecular techniques. The use of a Smart Cycler II TD (Cepheid) offers the possibility of further reducing the total time of this assay to as little as 45 minutes, as well as the potential for on-site testing of samples at a suspected outbreak site or at a port-of-entry. The assay was designed to complement morphological identification of thrips, not to replace it. Numerous different species of thrips have been found in trade, both major pest species and some quite obscure ones. Morphological expertise will continue to be required to deal with the complexity and range of identification work required. Nevertheless, the development of molecular tools of ever increasing speed and scope offer tools for the future that will further increase the efficiency of quarantine diagnostic laboratories. To that end, the cytochrome oxidase I gene from those thrips species and populations used to produce this assay have been sequenced and analyzed as the first step towards the production of a microarray protocol with the ultimate aim of producing an assay that offers simultaneous screening of numerous thrips species in a user-friendly format.

# Research article: Analysis of intra-plant *Tomato spotted wilt virus* infection distribution of *Nicotiana tabacum* L

**DOI:** 10.1673/031.007.2816

**Published:** 2007-05-04

**Authors:** S Connell, S Wright, A Csinos, R Gitaitis, C Nischwitz, M Stephenson, S Mullis

**Affiliations:** ^1^ Department of Plant Pathology, University of Georgia, P.O.Box 748, Tifton GA 31793.; ^2^ Crop and Soil Sciences, University of Georgia, P.O. Box 748, Tifton, GA 31793 spotwilt@uga.edu

*Tomato spotted wilt virus* (TSWV) has caused severe losses in tobacco in Georgia for approximately 15 years. Although much has been learned in that time, one aspect not clearly documented was the intra-plant distribution of TSWV. In previous studies, virus detected in roots was thought to be redistributed throughout the plant. This study presents a further evaluation of virus distribution in individual tobacco plants. At 8 weeks post transplanting during the 2005 growing season, root tissues from ninety non-symptomatic *N. tabacum* L. variety K-326 plants were tested for TSWV by DAS-ELISA (Agdia Inc., Elkhart, IN). Ten infected and 10 healthy plants were flagged for further analysis. Leaves were numbered sequentially from oldest to youngest on each plant and 100 mg tissue samples were collected bi-weekly for 3 wks and tested by DAS-ELISA. Within this period, TSWV was found more frequently in the oldest and youngest leaves. Plants with roots testing positive initially had the highest frequency of old and new leaf infections, whereas, plants initially testing negative primarily had TSWV in younger leaves with a subsequent lower frequency of TSWV in roots. Symptom expression did not necessarily follow this infection observation. It is not known if two separate inoculation periods, plant physiology, leaf size, virus titer or some other factor is responsible for this pattern. These data suggest that when testing for TSWV in tobacco, samples should be taken from multiple sources (leaves of various ages as well as roots) to increase the probability of detecting an infected plant.

# Research article: Australian acacia thrips

**Published:** 2007-05-04

**Authors:** BJ Crespi, DC Morris, LA Mound

**Affiliations:** CSIRO Entomology, Canberra, Australia. swmullis@uga.edu, Laurence.Mound@csiro.au

A single lineage of leaf-feeding Thysanoptera Phlaeothripinae has radiated solely on the plant genus Acacia in Australia into more than 250 species. The morphological diversity of these thrips is not closely correlated with their phylogenetic relationships as indicated by molecular studies using the genes CO1, 16S, EF1alpha and wingless. Molecular data indicates that behavioral character states are more strongly conserved than some morphological character states, the structural diversity within the kleptoparasitic thrips lineag being particularly high.

# Research article: Timing of application of Actigard and Admire for TSWV management in tobacco

**DOI:** 10.1673/031.007.2817

**Published:** 2007-05-04

**Authors:** AS Csinos, JE Laska, SW Mullis, MG Stephenson, SS LaHue, C Nischwitz

**Affiliations:** ^1^ University of Georgia, Dept. of Plant Pathology, Coastal Plain Experiment Station, Tifton, GA 31794; ^2^ University of Georgia, Dept. of Crop and Soil Science, Coastal Plain Experiment Station, Tifton, GA 31794; ^3^ University of Georgia, Field Research Services, Coastal Plain Experiment Station, Tifton, GA 31794 csinos@tifton.uga.edu; nisc5708@uidaho.edu

*Tomato spotted wilt virus* (TWSV) continues to be a serious problem in tobacco in Georgia. In 2003, about 40% of the tobacco crop in Georgia was infected. Both Actigard and Admire have shown to be effective against TWSV and reduce incidence. In this study the effect of different combinations and rates of Actigard and Admire as well as pre-plant and post-plant applications of the chemicals on TWSV incidence were tested. The treatments included an untreated control as well as a transgenic standard. A randomized complete block design was used consisting of single row plots that were repeated five times using K-326 fluecured tobacco. The plots were set up in fields at the Coastal Plain Experiment Station. Of the 16 different treatment combinations tested, twelve significantly reduced the percentage of symptomatic plants. The lowest percentages of symptomatic plants and of plants killed by TWSV were achieved by pre-plant treatments with Actigard and Admire plus post-plant treatments with Actigard that included an application 28 days after planting. Several treatments that included post-plant applications resulted in significantly higher yields compared to greenhouse application of Actigard and Admire alone. The two best treatments which consisted of pre-plant applications of Actigard and Admire and either one (28 days after planting) or three post-plant applications of Actigard were within 300lbs./A yield difference compared to the non-diseased transgenic standard and had over 1300lbs./A higher yield compared to the untreated control.

# Research article: Strategies for *Tospovirus* management: TSWV management in peanuts

**DOI:** 10.1673/031.007.2818

**Published:** 2007-05-04

**Authors:** AK Culbreath, JW Todd, SL Brown

**Affiliations:** The University of Georgia, Tifton Campus, P.O. Box 748, Tifton, GA 31793-0748 spotwilt@tifton.uga.edu

Tomato spotted wilt caused by thrips-vectored *Tomato spotted wilt virus* (TSWV) is a very serious problem in peanut (*Arachis hypogaea* L.) production in Georgia and the southeastern U.S. TSWV and the thrips, *Frankliniella fasca* and *Frankliniella occidentalis* that vector the virus present a difficult and complicated challenge from both epidemiological and disease management perspectives. Simply controlling the vectors with insecticides has seldom resulted in significant suppression of spotted wilt. Currently there is no single measure can provide adequate control of spotted wilt where severe epidemics occur. However, integrated management systems, using cultivars with moderate to high levels of field resistance, and suppressive chemical and cultural practices, have been developed and successfully deployed for minimizing losses to this disease. Adoption of some form of integrated management regime has been extensive and rapid in peanut producing areas of the U.S. where spotted is a problem. Although complete control of spotted wilt has seldom been achieved, integrated management practices have had a huge positive impact on peanut production in the presence of TSWV. The development of a spotted risk assessment index has aided greatly in relaying the importance of using an integrated approach for spotted wilt management. Natural field resistance appears to have the most potential for improving management of spotted wilt in peanut. New cultivars with high levels of field resistance have been developed and should greatly enhance management of spotted wilt as they are adopted. Breeding lines with even greater levels of field resistance than available in current cultivars have also been developed and are being increased for possible release as cultivars.

# Research article: The role of some intrinsic and extrinsic factors influencing take-off and landing

**DOI:** 10.1673/031.007.2819

**Published:** 2007-05-04

**Authors:** M Davidson, D Teulon

**Affiliations:** New Zealand Institute for Crop & Food Research Limited, Private Bag 4704, Christchurch, New Zealand

Thrips are weak fliers. After take off, thrips can be picked up by air currents flowing at strengths beyond their ability to fly against. Why do thrips launch themselves into such air currents? Likewise, what triggers them to drop out of the air currents? We have been investigating factors that may influence Western flower thrips' take off and subsequent landing. In a wind tunnel females starved for at least 4 hours were more likely to fly than those not starved. The age of female thrips did not affect the proportion to fly in a wind tunnel, however a larger proportion of older thrips (13 days old) landed on a yellow sticky trap than younger thrips (3 days old). The concentration of a volatile compound affected the proportion of thrips to fly in a wind tunnel, with fewer flying in the presence of 1 ml of p-anisaldehyde compared to 0.5 ml. Field trials were undertaken using water traps surrounding a central trap baited with a volatile compound known to elicit a positive response in New Zealand flower thrips or onion thrips. The highest numbers of thrips were recorded in the baited trap with decreasing numbers in unbaited traps positioned within 2 m downwind of the central trap. The implications of these results in relation to thrips' use of olfactory and visual cues, and thrips' dispersal are discussed.

# Research article: Development of a laboratory bioassay to test flight responses to semiochemicals in the western flower thrips, *Frankliniella occidentalis*

**DOI:** 10.1673/031.007.2820

**Published:** 2007-05-04

**Authors:** IAN Dublon, JGC Hamilton, WDJ Kirk

**Affiliations:** School of Life Sciences, Keele University, Staffordshire ST5 5BG, UK. i.a.n.dublon@keele.ac.uk

*Frankliniella occidentalis* (Pergande) is an established horticultural pest with a ubiquitous distribution. Its thigmotropic behaviour makes it difficult to control with insecticides and resistance is present in many strains. Two compounds, (*R*)-lavandulyl acetate and neryl (*S*)-2-methylbutanoate, isolated from the head-space of male *F. occidentalis* have been identified recently (Hamilton *et al*. 2005) and evidence thus far suggests that one of these compounds could be used for monitoring or mass trapping. The behavioral effects of these components on female and male *F. occidentalis* need to be investigated further in a laboratory environment. Y-tube bioassays only allow for the observation of a limited walking response. Alternative bioassays are therefore required to examine other responses, such as flight. Currently there are very few ways of bioassaying the flight response of thrips to specific semiochemicals within a laboratory without using a full-scale wind tunnel. Data are presented for the results of bioassays of the flight responses of adult female *F. occidentalis* to geraniol (*trans*-3,7-dimethyl-2,6-octadien-1-ol) using a novel twin-chamber moving-air bioassay. This compound is known to attract *F. occidentalis* in flight. It is hoped to use this working assay with the male-produced compounds. (Hamilton JGC. et al. 2005. *Journal of Chemical Ecology* 31: 1369–1379)

# Research article: Management of thrips using natural enemies

**DOI:** 10.1673/031.007.2821

**Published:** 2007-05-04

**Authors:** J Funderburk

**Affiliations:** North Florida Research and Education Center, University of Florida, 155 Research Road, Quincy, Florida 32351 USA. jefunderburk@mail.ifas.ufl.edu

Two groups of natural enemies are important biological controls of flower thrips in field crops. Species of *Orius* (Hemiptera: Anthocoridae) suppress rapidly growing populations of flower thrips, and they persist once the prey populations are suppressed. Species of *Thripinema* (Tylenchida: Allantonematidae) are parasites of flower thrips that render the female hosts sterile. Adult male and female hosts that are parasitized feed less, thereby reducing primary spread of tospoviruses. Parasitism results in suppression of subsequent larval populations which also prevents secondary spread of tospovirus. Both groups of natural enemies are important factors in the reduction of populations of flower thrips throughout the landscape. There are many other natural enemies of thrips, but their importance in affecting population dynamics is not adequately understood. These natural enemies offer potential for management of thrips and tospoviruses.

# Research article: Breaking of virus resistance in tomato associated with synergistic interaction between *Tomato chlorosis virus* and *Tomato spotted wilt virus*

**DOI:** 10.1673/031.007.2822

**Published:** 2007-05-04

**Authors:** E García-Cano, RO Resende, E Moriones

**Affiliations:** ^1^ Laboratorio Virología Vegetal, Estación Experimental “La Mayora”, CSIC, Málaga, Spain; ^2^ Dept. Biologia Celular, Universidade de Brasília, CEP 70.910-970, Brasília-DF, Brazil. rresende@unb.br

Tomato (*Lycopersicon esculentum*) is severely affected by viral diseases such as those caused by geminiviruses, tospoviruses, or criniviruses, which can result in significantly yield losses. Moreover, multiple viral infections are frequent found in nature with unpredictable pathological consequences. In fact, mixed infections between viruses may result in synergisms or antagonisms and can cause novel disease symptoms that could compromise virus control. In this work we have studied the possible interactions between the crinivirus (genus *Crinivirus*, family *Closteroviridae*) *Tomato chlorosis virus* (ToCV), and the tospovirus (genus *Tospovirus*, family *Bunyaviridae*) *Tomato spotted wilt virus* (TSWV), both viruses widely spread in the world. A tomato cultivar susceptible to both viruses, and a cultivar resistant to TSWV based on the *Sw-5* gene but susceptible to ToCV, were used for inoculations. Simultaneous or delayed inoculations were assayed. Symptoms expression and virus accumulation were monitored in plants. A dramatic synergism was observed between ToCV and TSWV in plants of the susceptible cultivar after the simultaneous inoculation of both viruses, leading to a rapid death of plants. However, this synergistic effect was not observed in delayed inoculations. Interestingly, results indicate that the presence of ToCV in *Sw-5*-resistant plants previous to TSWV inoculation result in TSWV-resistance breaking. Financial support: CAPES, CSIC, MECD, FINATEC.

# Research article: A Molecular identification for economically important thrips species

**DOI:** 10.1673/031.007.2823

**Published:** 2007-05-04

**Authors:** R Garza, R Ruiz, M Ciomperlik

**Affiliations:** USDA, APHIS, CPHST, Pest Detection and Diagnostic Management Laboratory, 22675 N. Moorefield Rd., Edinburg, TX 78541. roxanne.e.garza@aphis.usda.gov

*Scirtothrips dorsalis* Hood (Thysanoptera: Thripidae) is a new invasive pest to the Caribbean, and poses a significant threat to US agriculture and trade in the western hemisphere. This pest thrips has recently invaded the Caribbean islands of St. Vincent, St. Lucia, and Trinidad (Ciomperlik MA, Seal D. 2004. USDA APHIS PPQ, Technical Report). Since 1984, USDA-APHIS inspectors at various ports of entry have reported finding live *S. dorsalis* a total of 89 times from imported plant materials of 48 plant taxa (USDA 2003. Port Information Network, PIN-309: quarantine status database. USDA, APHIS, PPQ). *Scirtothrips dorsalis* is a polyphagous pest that is widespread in habitat ranging from temperate to tropical climatic regions in Pakistan, Japan and Australia (Mound and Palmer 1981). It is a pest of economic importance in citrus growing regions of Asia, where feeding can cause significant leaf deformation, fruit damage and yield reductions. *S. dorsalis* is an economically important pest of chili pepper, castor, cotton, onion and other crops with physical damage that can range from stunting and wilting of young leaves and shoots to total defoliation, and potentially heavy crop loss (Ananthakrishnan 1993. *Annual Review of Entomology* 38: 71–92; CABI/EPPO 1997. Quarantine Pests for Europe, 2nd Ed). Traditional morphological identification of *S. dorsalis* requires slide mounting of specimens and knowledge of distinct characters visible by microscopic examination. The accurate identification of pest species is fundamental in pest management programs. Molecular based methods provide a means for the accurate identification of species. A polymerase chain reaction-restriction fragment length polymorphism (PCR-RFLP) approach developed by Toda and Komazaki (2002, *Bulletin of Entomological Research* 92: 359–363) has been used to identify certain thrips species including *S. dorsalis* at our laboratory. In this study, PCR and DNA sequencing are being utilized to explore two regions of the mitochondrial gene, cytochrome oxidase I (COI). The DNA sequence data may provide information on the population genetic structure of S. dorsalis as well as its relationship to other thrips species. The DNA sequences will be analyzed to determine their potential for the development of species-specific molecular markers which will facilitate the design of real-time PCR assays. Real-time PCR will provide a fast and reliable method for the accurate identification of *Scirtothrips dorsalis*.

# Research article: Contemporary issues in *Tospovirus* molecular biology

**DOI:** 10.1673/031.007.2824

**Published:** 2007-05-04

**Authors:** TL German

**Affiliations:** Department of Entomology, University of Wisconsin, Madison, WI 53706, USA. tlg@entomology.wisc.edu

In 1984, Francki noted the similarity between *Tomato spotted wilt virus* (TSWV) and viruses in the family *Bunyaviridae*: a large group of membrane-bound, mostly arthropod-transmitted, animal-infecting viruses with tripartite negative-stranded RNA genomes. Data obtained in the last two decades elucidating the molecular biology of TSWV and the closely related *Impatiens necrotic spot virus* confirmed this observation and supplied the rational for the creation of the *Tospovirus* genus to include the plant-infecting, thrips-transmitted members of the family *Bunyaviridae*. Virions of TSWV, the type member of the family, are 80–120 nm pleomorphic particles with surface projections composed of two viral glycoproteins, GN and GC. The genome consists of three negative or ambisense ssRNAs designated S (2.9 kb), M (4.8 kb) and L (8.9 kb) with partially complementary terminal sequences that allow them to form a pseudocircular or panhandle conformation and likely serve as promoters for RNA synthesis. Each genomic RNA is encapsidated by multiple copies of the viral nucleocapsid protein (N) to form ribonucleocapsid structures (RNPs). The RNPs along with an estimated 20 copies of the L protein (RNA-dependent RNA polymerase) are enclosed in the viral membrane that is acquired during maturation at the Golgi. The ambisense S RNA encodes a 52.4 kDa nonstructural protein (NSs), in the viral (v) sense and the 29 kDa N protein in the viral complementary (vc) sense. The NSs protein is expressed from a v sense 1.7 kb subgenomic mRNA and the N protein from a 1.2 kb vc sense subgenomic RNA. The M RNA encodes a 33.6 kDa movement protein in the v sense and a 127.4 kDa precursor to GN and GC in the vc sense and relies on an expression strategy similar to that of the S RNA. The completely negative sense RNA contains a single open reading frame in the vc sense that is expressed from a nearly genome length mRNA. An additional 10–20 nucleotides of host origin are found at the 5′ ends of the viral N and NSs mRNAs providing evidence that TSWV uses the ends of host RNAs to acquire cap structures and direct transcription by means of a cap-snatching, typical of all segmented negative-sense RNA viruses. Details concerning the role of each viral protein in the infection cycle are beginning to emerge and recent work has suggested that, as expected, host proteins play a direct role as well. Future research directed towards understanding how proteins and nucleic acids interact to catalyze, regulate and target their activities will provide important new insights into the biology of these viruses.

# Research article: PCR Amplification of RNA of IYSV and TSWV recovered and stored on FTA cards

**DOI:** 10.1673/031.007.2825

**Published:** 2007-05-04

**Authors:** R Gitaitis, N Martinez, K Seebold, K Stevenson, H Sanders, S Mullis

**Affiliations:** ^1^ Univ. of Georgia, Tifton, GA;; ^2^ Lexington, KY,; ^3^ Univ. of KY, Lexington, KY USA. dronion@uga.edu

Tissues from onion leaves infected with Tomato Spotted Wilt Virus (TSWV), or Iris Yellow Spot Virus (IYSV) and from tobacco infected with TSWV were triturated in phosphate-buffered saline (PBS) and 100 *u*l of the homogenate were spotted onto Whatman FTA cards and allowed to dry. Discs (2–3 mm diam) were punched from the FTA card and the bound nucleic acids on the paper discs served as a template for RT-PCR. Amplification products were detected by gel electrophoresis through a 1.0% agarose gel containing ethidium bromide in a 0.04M Tris—acetate 0.001M EDTA (TAE) buffer. Basically constructed of filter paper impregnated with proprietary chemicals that are activated by biological fluids, FTA cards can be used for a variety of purposes ranging from the rapid extraction of nucleic acids from ephemeral samples immediately under field conditions to archiving DNA and RNA samples and storing them at room temperature. Furthermore, since viruses are inactivated, FTA cards can be used for transportation of genetic material without the need for special biohazard precautions or plant pest permits.

# Research article: Can transgenic virus resistance work in the real world — what about TSWV?

**DOI:** 10.1673/031.007.2826

**Published:** 2007-05-04

**Authors:** D Gonsalves

**Affiliations:** ARS/USDA Pacific Basin Agricultural Research Center, 99 Aupuni Street, Suite 204, Hilo, Hawaii 96720, USA. dgonsalves@pbarc.ars.usda.gov

Nearly 20 years ago, our laboratory embarked on developing virus resistant transgenic plants. The major viruses we focused on were papaya ringspot virus, tomato spotted wilt virus, and other vegetable viruses such as zucchini yellow mosaic, cucumber mosaic, and watermelon mosaic virus II. I will focus this talk on papaya ringspot virus and tospoviruses because they may present several lessons that will help to answer the question: Can Transgenic Virus Resistance Work in the Real World? The papaya work shows that transgenic virus resistance can work on a commercial scale and actually helped to save the papaya industry in Hawaii. Aside from the technical aspects, it also shows that timing and implementation of a transgenic product is important. One might say that the transgenic papaya was developed and commercialized before the ‘days of innocence’ with GMOs ended somewhere in 1998. To the present day, the papaya work also illustrates the type of forces or circumstances that affect the timely deployment of transgenic papaya outside of Hawaii. Relatively speaking, however, the papaya work did not shed as much light on the mechanism of transgenic virus resistance. In contrast, the work on tospoviruses did provide some insights on the mechanism and potential of transgenic tospovirus resistance. Given the range of crops that tospoviruses infect, one would expect that tospovirus resistance for commercial reasons might be a promising area of endeavor. However, I have not yet seen any signs that transgenic tospovirus resistance will be used commercially in the near future. Why is this? Is it because of technical difficulties? Is it because of the lack of will to do it? Or does the present GMO controversy suggest that the timing is not good for introducing tospovirus resistance to the real world? I will briefly discuss these aspects as they interrelate with my laboratory's experience on papaya ringspot virus and tospoviruses.

# Research article: Biorational control strategies for *Scirtothrips aurantii* that minimize non-target effects on arboreal and edaphic predatory mites

**DOI:** 10.1673/031.007.2827

**Published:** 2007-05-04

**Authors:** TG Grout

**Affiliations:** Citrus Research International, PO Box 28, Nelspruit, 1200 South Africa. tg@cri.co.za

South African citrus thrips (SACT), *Scirtothrips aurantii* Faure (Thripidae), is an indigenous pest of citrus in southern Africa and is often responsible for cosmetic damage to fruit that prevents the fruit from meeting export quality standards. Although the population density of SACT is largely determined by the availability of new, tender foliage (Gilbert MJ, Bedford ECG. 1998. In: Bedford ECG et al. editors. *Citrus Pests in the Republic of South Africa*. Second edition. 164–170. Republic of South Africa: Agricultural Research Council.), a complex of natural enemies helps to suppress the pest population. Some insect predators such as anthocorids, chrysopids and Thysanoptera are seldom abundant and levels of parasitism by *Goetheana incerta* Annecke are generally low. Predatory mites, and to a lesser extent, spiders, seem to be the most consistently abundant natural enemies both in the trees and in the soil below trees where SACT pupates. Little research has been conducted on non-target effects on spiders but recent research on the soil mite *Androlaelaps* sp. (Laelapidae) and earlier research on arboreal *Euseius* spp. (Phytoseiidae) (Grout TG, Richards GI.1992. *Experimental & Applied Acarology* 15: 199–204.; Grout TG *et al*. 1997. *Experimental & Applied Acarology* 21: 171–177) has enabled the selection of thripicides that are the least detrimental to these natural enemies. The efficacies of these and other biorational treatments were compared in the field with a view to finding the most effective but least disruptive thripicide. Application of insecticides via drip irrigation was also evaluated as a means of reducing non-target effects. Foliar treatments showing reasonable efficacy against SACT coupled with reduced mortality of predatory mites were: abamectin (0.00036% a.i.) plus oil (0.15%), spinosad (0.0072% a.i.) plus oil (0.3%), tartar emetic (0.199% a.i.) plus sugar (0.2%) and thiacloprid (0.0144% a.i.) plus oil (0.25%). Both dimethoate (36 g a.i./tree) and imidacloprid (2.1 g a.i./tree) showed promise as chemigation treatments.

# Research article: Ecologically based management systems for thrips in protected culture

**DOI:** 10.1673/031.007.2828

**Published:** 2007-05-04

**Authors:** KM Heinz, A Chau, S Arthurs

**Affiliations:** Department of Entomology; Texas A&M University, College Station, TX 77843. kheinz@ag.tamu.edu

Control tactics deployed against key pests for the foundation for a crop's pest management system around which all other control decisions must be coordinated. In the case of agriculture within protected systems, several species of thrips are commonly noted as the key pest for a particular cropping system. Management of these thrips species is built upon the best available research and education programs that address control foundations (proper identification of the key pest and how must it be controlled), stimuli for changing current management tactics (what forces are driving growers to move away from past practices), control combinations and their issues (what happens when two or more different kinds of control must be integrated), and techniques (what are the best available tool to accomplish the management goals). Protected culture provides a semi-permeable, replicated system for scientifically addressing the ecological and sociological factors influencing management systems. Examples from ornamentals and vegetables will be reviewed to identify factors leading to or impeding successful thrips management programs. A synthesis for systems-based approaches is developed for guiding future research objectives as well as in forming the foundation for novel thrips management programs.

# Research article: Strategies and issues to consider in traditional breeding for TSWV resistance in tomato

**DOI:** 10.1673/031.007.2829

**Published:** 2007-05-04

**Authors:** P Himmel

**Affiliations:** Seminis Vegetable Seeds, Woodland, California, USA. Phyllis.himmel@seminis.com

Tomatoes have been bred and selected for adaptation to specific growing areas for over 200 years and wild tomato species continue to be an important source of novel traits. *Tomato spotted wilt* causes yield losses in field and greenhouse tomato crops worldwide and unfortunately the efficacy of spray programs and cultural practices appears to be limited. Resistance to *Tomato spotted wilt* remains the most promising means of controlling this disease and can be found in *Lycopersicon pimpinellifolium* and *L. peruvianum*. Through wide interspecific crosses resistance has been introgressed into the cultivated tomato with good agronomic adaptability and commercial acceptability. Today, marker assisted selection for horticultural traits and disease resistance has streamlined the development of new commercial hybrids. The Seminis approach to breeding and selection for resistance, managing the resistance breaking isolates of *Tomato spotted wilt*, and the importance of combining resistance with key horticultural traits and marketing needs for a commercially successful product will be discussed.

# Research article: Can diel activity patterns in the western flower thrips be exploited as part of an IPM strategy?

**DOI:** 10.1673/031.007.2830

**Published:** 2007-05-04

**Authors:** ND Holmes, WDJ Kirk

**Affiliations:** School of life Sciences, Keele University, Staffordshire ST5 5BG, UK. n.d.holmes@keele.ac.uk

The western flower thrips *Frankliniella occidentalis* (Pergande) is a major worldwide horticultural pest. It damages plants by feeding and by the transmission of tospoviruses. Crop quality suffers as a result, leading to financial losses for growers. Chemical control of *F. occidentalis* is difficult because insecticide resistance is widespread. In addition, adults and larvae secrete themselves into small, tight spaces, limiting the effectiveness of current chemical treatments. Automated tracking and analysis software (EthoVision ver 3.1, Noldus Information Technology, Wageningen) has been used to record the diel behavior patterns of adult female thrips indicating when they walked, fed and retreated into small spaces. The resultant behavior profiles indicated the period of greatest activity during a 24-hour cycle when thrips were most likely to come into contact with a treatment, enhancing the effects of control procedures.

# Research article: Factors in low transmissibility of *Tomato spotted wilt virus* (TSWV) in asexual onion thrips, *Thrips tabaci* (Thysanoptera: Thripidae): effects of virus infection on longevity of thrips vector

**DOI:** 10.1673/031.007.2831

**Published:** 2007-05-04

**Authors:** T Inoue, T Sakurai

**Affiliations:** ^1^ United Graduate School of Agricultural Sciences, Iwate University, Morioka 020-8550, Japan; ^2^ Department of Biology and Environmental Sciences, National Agricultural Research Center for Tohoku Region, Morioka 020-0198, Japan. tsakurai@affrc.go.jp

Vector competence should be considered as the total transmission frequency during the potential transmission period from the onset of transmission to the vector death rather than the transmission rate for a certain period. The potential transmission period would be affected by vector longevity. If tospovirus infection has a negative effect on its vector longevity, the potential transmission period would shorten. In order to clarify the impact of infection of *Tomato spotted wilt virus* (TSWV) on the survival of two asexual *Thrips tabaci* populations, we compared i) the developmental rate, ii) the mortality before adult emergence and iii) the mortality during adult stage between virus exposed and non-exposed cohorts which were treated at young larval stage. No significant effects were observed in thrips developmental rate and the mortality rate during the developmental stage. However, the mortality trajectory showed that the age-specific mortality during adult stage was higher in virus exposed cohort than in non-exposed. We also estimated the potential transmission period from the mortality data and obtained the period of average 3.3 days in two tested populations. These results suggest that the potential transmission period shortened by TSWV infection may lead to low transmissibility of TSWV in asexual *T. tabaci* population.

# Research article: Overview of TSWV transmission by *Thrips tabaci* in Bulgarian tobacco fields

**DOI:** 10.1673/031.007.2832

**Published:** 2007-05-04

**Authors:** O Karadjova, V Krumov

**Affiliations:** Plant Protection Institute, Kostinbrod 2230, Bulgaria. oliakaradjova@abv.bg

Tobacco is a traditional field crop of great economic importance to Bulgaria. Tomato Spotted Wilt Virus (TSWV) was first reported in 1952 in Bulgaria and *Thrips tabaci* Lind was proved to be the only vector of the virus (Kovatchevsky I.1952. *Bulletin Rastitelna Zashtita* 3, *18–20;* Ivancheva-Gabrovska. 1984. *Studies on TSWV* (*Tomato Spotted Wilt Virus*) *in Bulgarian tobacco field*. Doctoral dissertation, Plovdiv, Bulgaria; Dimitrov A. 1991. *Biocenological studies on tobacco in Bulgaria and possibility for control of Thrips tabaci Lind. — the most important pest*. Doctoral dissertation, Plovdiv, Bulgaria; Hristova D. *et. al*. 2001. *Journal of Phytopathology* 149, 1–5). Two different *Th*. *tabaci* populations — arrhenotokous (Th.t.1) and thelotokous (Th.t 2) were tested for their ability to transmit two TSWV isolates (BR-01 and GD from tobacco). DAS-ELISA was used to confirm the infection in individual thrips. The transmission efficiency of adult *Th. tabaci* was tested on petunia leaf discs (Wijkamp, I. 1995. *Virus-vector relationships in the transmission of tospoviruses*. Ph. D thesis, Wageningen, the Netherlands). Thrips and TSWV incidences were investigated in Bulgarian tobacco fields for a period of 50 years and the climatic conditions for every year were taken into account. The data analysis shows that TSWV epidemic has a 5–6 year interval of occurrence. The main outbreaks of TSWV were observed country wide in 1969 (57 %) and 1983 (53 %). Although the climatic conditions through these two years were favorable for both development of *Th. tabaci* and viral infection, during the remaining years no relation between TSWV epidemics and thrips infestation has been established. For example in 1972 there was 35% thrips infestation of tobacco plants, but TSWV infection was only 5%. The opposite phenomenon occurred in 1960 — 9% of thrips infestation corresponded to 23% of TSWV infection. Positive ELISA values were found only in the arrhenotokous population (Th.t.1). The average ELISA values were higher for TSWV —GD isolate, male individuals (OD = 0.377). Comparative laboratory studies for measuring of TSWV transmission efficiency of arrhenotokous and thelytokous populations have been conducted. Our investigation showed that the arrhenotokous population of *Th. tabaci* from tobacco is an efficient vector of TSWV (range from 2.08 to 39.58 %). The transmission rates of male specimens were higher than those of female ones. TSWV isolate GD from tobacco was transmitted more efficiently than BR-01. The telotokous population was not able to transmit both tested isolates.

# Research article: Transmission of TSWV in greenhouse crops in Bulgaria

**DOI:** 10.1673/031.007.2833

**Published:** 2007-05-04

**Authors:** O Karadjova, V Krumov, P Nikolov

**Affiliations:** ^1^ Plant Protection Institute, 2230 Kostinbrod, Bulgaria; ^2^ National Service for Plant Protection, 55 Hr. Botev Blvd., Sofia, Bulgaria. oliakaradjova@abv.bg

Since the Western Flower Thrips (*Frankliniella occidentalis Perg*.) was first reported in Bulgaria in 1991, it has become one of the most important greenhouse crop pests (Trenchev G, Karadjova O. 1992. *Plant Protection Journal* 3: 26–30; Karadjova O *et al*. 2003. *Acta Entomologica Bulgarica*. 9: 22–29). The first TSWV epidemic was recorded in 1993 in many greenhouses across the country (Hristova D *et al*. 2001. *Journal of Phytopathology* 149: 1–5). Until now, TSWV is the only tospovirus that has been found on vegetables and ornamental crops in Bulgaria, although INSV has been found on imported planting material especially from Israel and the Netherlands. The absence of INSV Tospovirus, also in ornamentals, indicates that at present the tospovirus population in Bulgaria is indigenous and not contaminated with strains from Western Europe (Hristova *et al*. 2001). Two different *F. occidentalis* populations (F.occ. 1 and F.occ. 2) and three TSWV isolates (BR-01, GD from tobacco and KR from greenhouse tomato) were used in this investigation. DAS-ELISA was used to confirm infection in individual thrips. The transmission efficiency of adult *Th. tabaci* was tested on petunia leaf discs (Wijkamp I. 1995. *Virus-vector relationships in the transmission of tospoviruses*. Ph. D thesis, Wageningen, the Netherlands). Positive ELISA values were detected in both F. occidentalis populations. The highest percent TSWV positive thrips was established for the combination F. occ. 2 - TSWV KR (Bulgarian greenhouse isolate from tomato). The lowest ELISA values were detected for F.occ.1 - TSWV isolate from tobacco (GD). Insect transmission tests were conducted with the same populations. Our tests demonstrated successful transmission of TSWV by both *F. occidentalis* populations. The highest transmission rate was observed for the combination F.occ. 2 — KR isolate (52.08%). Transmission efficiency of F.occ. 1 — TSWV BR-01 reached 27.08% and the lowest transmission was observed in F.occ. 1 — GD (6.25%). Results of virus transmission to leaf discs were correlated to the readings in ELISA.

# Research article: Thrips and *Tospovirus* management: an overview

**DOI:** 10.1673/031.007.2834

**Published:** 2007-05-04

**Authors:** G Kennedy

**Affiliations:** Department of Entomology, North Carolina State University, Raleigh, NC USA. george_kennedy@ncsu.edu

This presentation will provide a brief overview of recent advances in our understanding of thrips population dynamics and *Tomato spotted wilt virus* (TSWV) epidemiology as they relate to currently available options for TSWV management. The constraints on TSWV management imposed by modern cropping systems will be discussed in an attempt to identify the essential attributes of viable management options for TSWV.

# Research article: Do thunderstorms affect the flight behavior of thrips?

**DOI:** 10.1673/031.007.2835

**Published:** 2007-05-04

**Authors:** WDJ Kirk

**Affiliations:** School of Life Sciences, Keele University, Staffordshire ST5 5BG, UK. w.d.j.kirk@keele.ac.uk

Across northern Europe, thrips are popularly associated with thunderstorms. Although they fly mainly in fine weather, there is indirect evidence that they land when thunderstorms are near. The effect is most obvious for abundant species, such as cereal thrips. One hypothesis is that thrips cannot fly in rapidly changing electric fields that occur near thunderstorms, so they are forced to land (Kirk WDJ. 2004. *Phytopathologica et Entomologica Hungarica* 39: 131–136). Experiments are needed to test the effects of electric fields on the flight behavior of thrips.

# Research article: The chemical language of thrips

**DOI:** 10.1673/031.007.2836

**Published:** 2007-05-04

**Authors:** WDJ Kirk

**Affiliations:** School of Life Sciences, Keele University, Staffordshire ST5 5BG, UK. w.d.j.kirk@keele.ac.uk

There is increasing evidence that chemical communication plays a major role in many aspects of the life of the western flower thrips *Frankliniella occidentalis* (Pergande) (Thysanoptera: Thripidae). This now includes research on an alarm pheromone produced by larvae and an aggregation pheromone produced by adult males, as well as research on responses to plant volatiles and related compounds. Since this species has only become the focus of research because it is a major pest, it is likely that chemical communication also plays a major role in the biology of many other species of thrips. Thus, a vast research field is opening up. Current developments in the chemical ecology of the western flower thrips and other thrips are reviewed, and possible future research directions and applied prospects are considered.

# Research article: *Wolbachia* — protected or not protected is the question

**DOI:** 10.1673/031.007.2837

**Published:** 2007-05-04

**Authors:** S Kumm, G Moritz

**Affiliations:** Martin-Luther-University Halle-Wittenberg, Developmental Biology, Domplatz 4, 06108 Halle, Germany. sandra.kumm@zoologie.uni-halle.de

The last two decades revealed *Wolbachia* as one of the most widespread bacteria among arthropods. Bacteria of this genus are known to cause diverse reproductive manipulations in their hosts including cytoplasmic incompatibility, male killing, parthenogenesis, and feminization. In thrips females are usually diploid and males are haploid. The most common form of reproduction is arrhenotoky, but thrips also often reproduce by thelytoky. A couple of species are known to reproduce by thelytoky in some geographical areas, whereas the sex ratio values indicate arrhenotoky in other areas. *Wolbachia* was discovered in the thelytokous thrips species, *Heliothrips haemorrhoidalis* and *Hercinothrips femoralis* (Pintureau B *et al*. 1999. *Annales de la Societe Entomologique de France* 35: 416–420) and was proven to be responsible for thelytoky in *Franklinothrips vespiformis* (Arakaki N. *et al*. 2001. *Proceedings of the Royal Society of London*, *Series B* 268: 1011–1016). So far only thelytokous thrips species were tested. Therefore, in the present study we screened thrips species displaying different reproduction modes for the presence of *Wolbachia* using 16S rDNA and ftsZ gene primer. The thelytokous species *Hercinothrips femoralis* and *Parthenothrips dracaenae* possess *Wolbachia* and in *H. femoralis Wolbachia* was shown to be the reason for thelytokous reproduction since males were produced after antibiotic treatment. Males were also observed to copulate with females and spermathecae of females were filled with sperm. But it was not possible to generate an arrhenotokous line in *H. femoralis*. The arrhenotokous species *Echinothrips americanus*, *Suocerathrips linguis*, and *Gynaikothrips ficorum* also harbour *Wolbachia*, but the bacterium was not detected in *Frankliniella occidentalis* and *Thrips tabaci*. Neither thelytokous nor arrhenotokous populations of the latter species possess *Wolbachia*. Sequence analysis of three species, *H. femoralis*, *P. dracaenae*, and *S*. *linguis*, by direct sequencing confirmed the presence of *Wolbachia*, whereby all tested species are infected with different *Wolbachia* types. Antibiotic and heat treatment of thelytokous *T. tabaci* did not lead to male production and therefore bacteria are likely to exclude as being responsible for thelytoky in this species. Thus, it can be concluded that probably two different forms of thelytoky exist in Thysanoptera, one induced by microorganisms like in *H. femoralis* and a non-revertible (not induced by micro-organisms) like in *T. tabaci*. All positive tested species are no tospovirus vectors and species which do not possess the bacterium are known to act as tospovirus transmitters. Maybe there is some mechanism which protects tospovirus vectors from getting infected with *Wolbachia* or the opposite way around. It should be also proofed if and what influence *Wolbachia* has on arrhenotokous thrips species. Perhaps they also exhibit some kind of defence reaction against the bacterium suppressing the manipulation of reproduction.

# Research article: The N protein of *Tomato spotted wilt virus:* the putative elicitor of a hypersensitive response (HR) in *Capsicum chinense*

**DOI:** 10.1673/031.007.2838

**Published:** 2007-05-04

**Authors:** FA Lovato, T Nagata, AC Ávila, RO Resende, AK Inoue-Nagata

**Affiliations:** ^1^ Departamento de Biologia Celular, UnB, Brasilia, DF; ^2^ Embrapa Hortaliças, Brasília, DF. alicenag@cnph.embrapa.br

*Tomato spotted wilt virus* (TSWV) causes a serious disease in plants worldwide. The three genomic RNA's of TSWV encode three structural proteins (L, G1/G2 and N) and two non-structural proteins (NSM and NSS). Reaction to TSWV inoculation resembling the hypersensitive response (HR) has been previously reported in *Capsicum chinense* ‘PI152225’ and ‘PI159236’. The aim of this study was to identify the TSWV elicitor(s) protein(s) related to this HR-like response in *C. chinense*. For this purpose, the N, NSM and NSS genes of TSWV were cloned into the binary vector pgR107, based on *Potato virus X* (PVX) expression system via agroinoculation. Recombinant agrobacterium colonies transformed with the N, NSM and NSS gene constructs were inoculated into *C. chinense* ‘PI159236’. After 10–15 days, typical symptoms of PVX appeared in the upper leaves of plants inoculated with the constructions PVX+N, PVX+NS_M_ and PVX+NS_S_. ELISA and Western blot confirmed the N, NSM and NSS protein expression in inoculated plants. HR-like necrotic lesions were seen only in ‘PI159236’ infected with the construct PVX+N. The symptomatic leaves showed typical leaf abscission after 30–35 days. This result indicated that the N gene of TSWV is possibly the elicitor of the HR-like response in *C. chinense*. In addition, leaves of *C. chinense*, *D. stramonium* and *N. benthamiana* inoculated with the construction PVX+NSS showed severe necrotic symptoms, suggesting an increase of PVX symptom expression caused by a possible synergism of PVX with the NSS protein. Financial Support: CNPq, Embrapa, UnB, FINATEC.

# Research article: The thrips complex associated with onion in Colorado

**DOI:** 10.1673/031.007.2839

**Published:** 2007-05-04

**Authors:** L Mahaffey, W Cranshaw, H Schwartz

**Affiliations:** Department of Bioagricultural Sciences and Pest Management Colorado State University, Ft. Collins, CO 80523. linda.mahaffey@colostate.edu

Thrips problems affecting onions in Colorado have increased greatly in recent years because of: 1) difficulties in control due to development of insecticide resistance; and 2) emergence of thrips-vectored Iris yellow spot virus as a new disease of the crop. In response, a wide range of studies are being conducted at Colorado State University to better understand seasonal biology of thrips associated with onion and identifying effective pest management practices. Onion thrips, Thrips tabaci, is the predominant species in all production areas followed by western flower thrips, F. occidentalis and F.schultzei. Over 95% of all thrips are T. tabaci in most areas, but F. occidentalis was present in high percentage (ca. 24%) in southwestern Colorado. Introduction of thrips on transplants grown in California, Arizona and Texas is a significant source of thrips with 57% (8/14) and 95% (21/22) of samples infested in 2004 and 2005, respectively. Thrips tabaci predominated but other species present were F. ewarti, F. occidentalis, and Scirothrips longipennis. The ratio of Thrips tabaci to Frankliniella spp. did not shift in fields with repeated history of pyrethroid use (lambda-cyhalothrin).

# Research article: Molecular characterization of resistance-breaking strains of *Tomato spotted wilt virus* (*Tospovirus, Bunyaviridae*) infecting resistant pepper carrying the *Tsw* gene

**DOI:** 10.1673/031.007.2840

**Published:** 2007-05-04

**Authors:** P Margaria, M Turina

**Affiliations:** Istituto di Virologia Vegetale, CNR, Torino 10135, Italy. m.turina@ivv.cnr.it

*Tomato spotted wilt virus* (TSWV) (*Tospovirus, Bunyaviridae*) causes one of the most devastating diseases of pepper (*Capsicum* sp.) worldwide. The most effective and environmentally sound control strategy for this virus relies on the availability of resistant pepper cultivars. All known resistant cultivars possess a single dominant resistance gene, *Tsw*. Following TSWV infection, *Tsw* induces a hypersensitive-like reaction (HR), characterized by local necrotic lesions, that prevents the spread of the virus from the point of entry. Recently, naturally occurring resistance-breaking TSWV strains have been identified, causing major concern. We are utilising a collection of RB-TSWV strains to identify the specific genetic determinant that allows the virus to overcome the *Tsw* gene in *Capsicum*. As a reverse genetic approach is not feasible for minus-strand genome plant viruses such as TSWV, we set up a series of reassortment experiments between the wild-type strain Br01 and RB- TSWV strains. Our results confirm that the S RNA, which encodes both the nucleocapsid (N) and a nonstructural protein (NSs), carries the genetic determinant responsible for the breakdown of *Tsw* resistance. Sequence comparison between full-length S RNA segments of wild type and RB strains allows us to speculate about the specific genetic determinant of the “avirulence” factor.

# Research article: Natural infections of *Tomato spotted wilt virus* (TSWV) found in weeds around tobacco fields in Georgia

**DOI:** 10.1673/031.007.2841

**Published:** 2007-05-04

**Authors:** N Martinez-Ochoa, SW Mullis, AS Csinos, TM Webster, P Bertrand

**Affiliations:** ^1^ Dept. of Plant Pathology, University of Georgia, Tifton GA 31793 U.S.A.; ^2^ CPMRU, USDA-ARS, Tifton GA 31793 U.S.A. nmartinez8@alltel.net

*Tomato spotted wilt virus* (TSWV) is now endemic to the southeastern United States, where it affects major agricultural crops such as tomato, pepper, tobacco, peanut and numerous ornamentals as well. Due to milder winters and longer humid and warm summers in Georgia, there are favorable conditions for a succession of plant species that could serve as tospovirus reservoirs and more importantly, for a place for the thrips vectors to reproduce throughout the year. A three-year survey was conducted in nine Georgia tobacco fields to determine the weed species that were naturally infected with TSWV. Samples were taken on a monthly basis from most weed types at each location, and leaf and root tissues were tested together using DAS-ELISA (Agdia Inc.). In the winter and spring, *Oenothera laciniata* Hill (cutleaf evening primrose), *Gnaphalium pensylvanicum* Wild. (wandering cudweed), *G. purpureum* L. (purple cudweed), *Eupatorium capillifolium* (Lam.) Small (dogfennel), *Lepidium virginicum* L. (Virginia pepperweed), and *Geranium carolinianum* L. (Carolina geranium), were among the wild plant species most commonly infected with TSWV. In the summer and fall, the most common species found testing positive for TSWV were *Jacquemontia tamnifolia* (L.) Griseb. (smallflower morningglory), *Richardia scabra* L. (Florida pusley), *Portulaca pilosa* L. (broadleaf pink purslane), *Desmodium tortuosum* (Sw.) DC. (Florida beggarweed), *Amaranthus retroflexus* L. (redroot pigweed), and *Wahlenbergia marginata* (Thunb.) A. DC. (southern rockbell). There were a few plant species that did not test positive for TSWV during the entire survey period, and examples of those were *Viola fafinesquii* Greene (field pansy), *Cardamine hirsuta* L. (hairy bittercress), *Diodia teres* Walt (poorjoe), *Veronica arvensis* L. (corn speedwell), and *Phylotacca americana* L. (common pokeweed). Focus in follow-up surveys is being aimed at a select group of the most commonly TSWV-infected weeds found that are also reported as being reproductive hosts for the thrips vectors. In addition to weather and thrips vector populations, it is expected that seasonal incidences of TSWV in their weed reservoirs might be useful in predicting TSWV levels when planting susceptible field crops.

# Research article: Host ranges and potential as pests: ways of some thrips species in southern areas

**DOI:** 10.1673/031.007.2842

**Published:** 2007-05-04

**Authors:** R Marullo

**Affiliations:** Dipartimento di Agrochimica ed Agrobiologia, Università degli Studi Mediterranea di Reggio Calabria, Italy. rmarullo@unirc.it

Data on the host plant ranges for thrips species are often scanty, and are commonly derived from adult specimens that may have dispersed from their breeding sites. Because of this, there are many difficulties to present a comprehensive list of the host plants for most Thysanoptera species. Despite this, different thrips species exploit the available plants to varying extents, such that some may be considered as monophagous, oligophagous or even polyphagous. Polyphagous thrips species are more likely to be pest species than monophagous or oligophagous species (Moritz G *et al*. 2004. *Pest thrips of the world: an identification and information system using molecular and microscopical methods*). Also polyphagous species sometimes produce localized strains with a strong attachment to a particular plant species. Polyphagy and host range might be related to availability of particular compounds in the host plants (Terry I. 1997. In: *Thrips as Crop Pests*, 65–118), or such thrips species might be unusually flexible in their feeding behaviour. However, host plant associations sometimes vary between localities, and a few thrips species are known to exhibit remarkable host shifts (Mound LA. 2005. *Annual Reviews of Entomology* 50: 247–269), such that they become pests on plants unrelated to their “natural” hosts. In the present talk two species from southern Italy are considered that have remarkably disjunct host relationships: *Neohydatothrips gracilicornis* (Williams) on *Vicia* (Fabaceae) and *Pinus* (Pinaceae) (Marullo R. 1990. *Redia* 73 :223–228), and *Drepanothrips reuteri* Uzel on *Vitis* (Vitaceae) and *Quercus* (Fagaceae). In contrast the polyphagous species *Heliothrips haemorrhoidalis* (Bouchè) is expanding as a pest in forest areas, whereas it is no longer a severe pest of *Citrus* on which crop it is completely supplanted by *Pezothrips kellyanus* (Bagnall) in the intensive citrus southern orchards (Navel orange, lemon and bergamot). Here, for each thrips species, the main features of the life history, based on field data, and the “status” on the new host plants are focused and discussed. *N.gracilicornis* breeds on plants of wild *Vicia* spp., and also other Fabaceae, larvae develop mainly in the flowers of these host plants and pupate into the soil. The species is univoltine and overwinters as an adult living in litter or amongst plant debris in the soil. Adults can feed on Pinus needles during autumn and winter, but damage symptoms are induced only on weakly growing plants. Instead, both *D.reuteri* and *H.haemorrhoidalis* have *Quercus* as host plant, on which they breed and can have a few generations per year.

# Research article: Effect of verbena as a trap crop on incidence of *Tomato spotted wilt virus* in chrysanthemums

**DOI:** 10.1673/031.007.2843

**Published:** 2007-05-04

**Authors:** S Matsuura, S Hoshino, H Koga

**Affiliations:** ^1^ Hiroshima Prefectural Agriculture Research Center, Higashi-hiroshima, Hiroshima 739-0151, Japan.; ^2^ Taiyo Corporation Co. Ltd, Higashi-yodogawa, Osaka 533-0023, Japan s-matsuura81314@pref.hiroshima.jp

The effect was investigated of verbena as a trap crop on the occurrence of western flower thrips, *Frankliniella occidentalis*, and the incidence of TSWV in chrysanthemums. Verbena cvs Pink Parfait and/or Fancy Parfait were cultivated alongside chrysanthemum cv. Jimba in a greenhouse at the proportion of 17–25% of chrysanthemum plants. Verbena cultivation suppressed the occurrence of western flower thrips on chrysanthemum until flower bud initiation by attracting vector thrips, and reduced TSWV incidence in the chrysanthemums up to flowering to 13–18% of untreated controls. We found that linalool oxide (pyran), an attractive volatile for vector thrips (Hooper *et al*. 1999: Pestic Sci 55:660–662), was mainly produced by the flower of verbena cv. Fancy Parfait and the ratio of *cis* and *trans*-type was 1 : 5. Our results suggest that cultivation of verbena as a trap crop suppresses TSWV incidence in the chrysanthemums up to the flowering stage and has the potential to be utilized as a control measure in IPM programs.

# Research article: Expression of a viral polymerase-bound host factor turns human cell lines permissive to a plant- and insect-infecting virus

**DOI:** 10.1673/031.007.2844

**Published:** 2007-05-04

**Authors:** RB Medeiros, J J Figueiredo, RO RO Resende, AC Avila

**Affiliations:** ^1^ Dept. Fitopatologia and 2Biologia Celular, Universidade de Brasília, Brasília-DF, Brazil.; ^2^ Dept. Biologia Celular, Universidade de Brasília, Brasília-DF, Brazil; ^3^ Centro de Hortaliças, Empresa Brasileira de Pesquisa Agropecuária, Brasília-DF, Brazil. demed002@umn.edu

*Tospoviruses* are the only plant-infecting members of the *Bunyaviridae* family of ambisense ssRNA viruses. *Tomato spotted wilt* tospovirus (TSWV), the type-member, also causes mild infection on its main insect vector, *Frankliniella occidentalis*. Herein, we identified an *F. occidentalis* putative transcription factor (FoTF) that binds to the TSWV RNA-dependent RNA polymerase and to viral RNA. Using in vitro RNA synthesis assays, we show that addition of purified FoTF improves viral replication, but not transcription. Expression of FoTF deletion mutants, unable to bind the RNA-dependent RNA polymerase or viral RNA, blocks TSWV replication in *F. occidentalis* cells. Finally, expression of FoTF wild-type turns human cell lines permissive to TSWV replication. These data indicate that FoTF is a host factor required for TSWV replication in vitro and in vivo, provide an experimental system that could be used to compare molecular defense mechanisms in plant, insect, and human cells against the same pathogen (TSWV), and could lead to a better understanding of evolutionary processes of ambisense RNA viruses. Financial Support: CNPq (CBAB), Embrapa, UnB, FINATEC.

# Research article: Isolation of entomopathogenic fungi and their pathogenicity against the Chili Thrips, *Scirtothrips dorsalis*

**DOI:** 10.1673/031.007.2845

**Published:** 2007-05-04

**Authors:** G Mikunthan, M Manjunatha

**Affiliations:** ^1^ Dept. Agricultural Biology, Faculty of Agriculture, University of Jaffna, Jaffna, Sri Lanka,; ^2^ Department of Entomology, College of Agriculture, Shimoga 577201, Karnataka, India gunamiku@sify.com

The Chili Thrips, *Scirtothrips dorsalis* (Thysanoptera: Thripidae) is considered as a polyphagous insect pest causing severe upward leaf curl in chili (*Capsicum annuum* L.). This dreaded pest is being controlled by frequent use of selective and expensive systemic insecticides however, resistance developed in these insects limits the long term application and dependency on these chemicals in near future. Biological control is a viable alternative strategy that can be incorporated in to the integrated management of thrips to overcome this problem. However a sound knowledge of the natural enemy complex is very essential as an opted alternate choice to chemicals. Among the natural enemy complex, pathogens regulating the population of *S. dorsalis* have not been extensively studied. Added to this, there are no reports available on fungi infecting *S. dorsalis*. An attempt was made to isolate entomopathogenic fungi from chili fields and to evaluate their pathogenicity against *S. dorsalis*. A roving survey was conducted during 2002 to collect soil and foliage samples from chili fields representing ten agroclimatic zones of the Karnataka State of India. Entomopathogenic fungi were isolated from soil using a soil bait method (Zimmerman G. 1986. *Journal of Applied Entomology* 102: 213–215; Parker BL *et al*.1996. In: Tospoviruses and thrips, *ISHS Acta Horticulturae* 431: 521–534) and from infected cadavers collected from chili leaves. Larvae of *Tribolium castaneum* (Coleoptera: Tenebrionidae) and wax moth, *Galleria melonella* (Lepidoptera: Galleridae) were used as soil baits. Thirteen fungal isolates were recovered from soil using larvae of *T. castaneum* and *G. melonella*. However none of them were able to infect on any stages of *S. dorsalis*. Three fungi *viz. Fusarium* sp. isolate GM15 (ARSEF 7381), *Neozygites floridana* and *Penicillium* sp. were isolated from diseased cadavers of larvae of *S. dorsalis* and *Fusarium semitectum* (ARSEF 7233) from larvae of *S. dorsalis*, active stages of *Polyphagotarsonemus latus* (Acarina: Tarsonemidae) and nymphs and adults of *Aphis gossypii* (Hemiptera: Aphididae) from chili leaves. A local isolate of *Metarhizium anisopliae* was also found infective to adults of *S. dorsalis* and caused 13 per cent mortality. The isolation of entomopathogenic fungi from *S. dorsalis* and *Fusarium* species from *S. dorsalis*, *P. latus* and *A. gossypii* are new records. Koch's postulates confirmed the infectivity of *Fusarium* isolates on *S. dorsalis* and the pathogenicity studies revealed that *F. semitectum* and *Fusarium* sp. isolate GM15 caused 86 and 76 per cent mortality of larvae of *S. dorsalis*, respectively. *N. floridana* caused 82 per cent mortality. These results show the potential use of entomopathogenic fungi in controlling *S. dorsalis*. The identity of the *Fusarium* isolates was confirmed by Dr. D. M. Geiser, Pennsylvania State University and *Neozygites floridana* by Dr.R.A.Humber of USDA-ARSEF, Collection of Entomopathogenic Fungal Cultures.

# Research article: Thysanoptera fauna of Shiraz and vicinity

**DOI:** 10.1673/031.007.2846

**Published:** 2007-05-04

**Authors:** K Minaei, M Alichi

**Affiliations:** ^1^ Plant Protection Department, College of Agriculture, Tehran University, Karaj-Iran.; ^2^ Plant Protection Department, College of Agriculture, Shiraz University, Iran kminaei@ut.ac.ir

During investigations on the thysanopteran fauna of Shiraz (Fars province, south west of Iran) and its vicinity in 1999–2002, forty-two species from 5 families and 23 genera were collected. They are listed according to their families as follows: Family Adiheterothripidae- *Holarthrothrips josephi* Bhatti; Family Aeolothripidae-*Aeolothrips collaris* Priesner, *Aeolothrips intermediu s Bagnall, Aeolothrips tenuicornis bagnall, Aeolothrips versicolor* Uzel, *Orothrips priesneri* (Titschack), *Rhipidothrips gratiosus* Uzel; Family Melanthripidae- *Melanthrips fuscus* Sulzer, *Melanthrips rivnayi* Priesner, *Melanthrips separandus* Priesner; Family Phlaeothripidae: *Cephalothrips coxalis* Bagnall, *Compsothrips albosignatus* (Reuter), *Haplothrips aculeatus* (Fabricius), *Haplothrips flavitibiae* Williams, *Haplothrips niger* (Osborn), *Haplothrips reuteri* Karny, *Haplothrips subtilissimus* (Haliday), *Haplothrips tolerabilis* Priesner, *Haplothrips tritici* Kurdjumov, *Liothrips pragensis* Uzel, *Megathrips flavipes* (Reuter), *Pseudocryptothrips meridionalis* Priesner; Family Thripidae- *Agrostothrips meridionalis* (Bagnall), *Anaphothrips obscurus* (Muller), *Anaphothrips sudanensis* Trybom, *Chirothrips kurdistanus* Zur Strassen, *Chirothrips pallidicornis* Priesner, *Frankliniella intonsa* (Trybom), *Frankliniella pallida* Uzel, *Frankliniella tenuicornis* (Uzel), *Neohydatothrips tadzhicus* (Pelikan), *Odontothrips confusus* Priesner, *Retithrips syriacus* (Mayet), *Scirtothrips mangifera* Priesner, *Scolothrips latipenis* Priesner, *Sitothrips arabicus* Priesner, *Tenothrips discolor* (Karny), *Tenothrips frici* (Uzel), *Thrips major* Uzel, *Thrips meridionalis* (Priesner), *Thrips minutssimus* Linnaeus, *Thrips tabaci* Lindeman. The species *Aeolothrips versicolor*, *Haplothrips subtilissimus* and *Scolothrips latipenis* are predators of small arthropods and spider mites respectively. *Compsothrips albosignatus*, *Megathrips flavipes*, and *Pseudocryptothrips meridionalis* feed on fungal spores. The other species are considered phytophages or pollen-feeders. (Minaei K. 2000. *Thysanoptera fauna of Shiraz and vicinity*. M. Sc. Thesis, Shiraz University, Shiraz, Iran; Minaei K et al. 2001. *Iran Agircultural Rearch* 20: 53–66)

# Research article: Pest thrips in Iran: An introduction

**DOI:** 10.1673/031.007.2847

**Published:** 2007-05-04

**Authors:** K Minaei, P Azemayeshfard

**Affiliations:** Plant Protection Department, College of Agriculture, Tehran University, Karaj-Iran. kminaei@ut.ac.ir

About 161 thrips species from 5 families and 60 genera have been recorded in Iran. However information related to the species composition of thrips populations and the significance of species as potential phytophages or carnivores has been given less attention, and much of the published data on host plant ranges of thrips is inaccurate in our country. At present, eight species are considered to damage cultivated plants in Iran: *Anaphothrips obscurus* (Müller), *Drepanothrips reuteri* Uzel, *Haplothrips tritici* (Kurdjumov), *Pseudodendrothrips mori* (Niwa), *Retithrips syriacus* (Mayet), *Rubiothrips vitis* (Priesner), *Thrips flavus* Schrank and *T. tabaci* Lindeman. Furthermore, there are some superficial reports on thrips pests. *Thrips tabaci* is the most damaging insect pest of onions in Iran. This species and wheat thrips (*Haplothrips tritici*) are the two that get most attention from Iranian entomologists due to their economic importance. The presence of the introduced thrips, *Frankliniella occidentalis* (Pergande) has been confirmed but there is no information on its pest status and distribution. Four known vector species of Tospoviruses are recorded in Iran: *Frankliniella intonsa*, *F. occidentalis*, *F. schultzei*, and *Thrips tabaci.Tomato spotted wilt virus* (TSWV) has been recorded in Iran, and transmission of cineraria (*Senecio* sp.) isolate of TSWV has been confirmed by *Thrips tabaci*. Recently a new *Tospovirus* species infecting tomato namely Tomato fruit yellow ring virus has been recorded in Iran. (Bhatii JS, et al.. 2003. *Proc. Natnl. Symp. Frontier Areas Ent. Res*., Pp. 668–669; Ghotbi T et al. 2005. *Plant Disease* 89: 425–429).

# Research article: Anatomy and development of thrips relative to *Tospovirus* acquisition

**DOI:** 10.1673/031.007.2848

**Published:** 2007-05-04

**Authors:** G Moritz

**Affiliations:** Martin-Luther-University Halle-Wittenberg, Developmental Biology, Domplatz 4, 06108 Halle, Germany. gerald.moritz@zoologie.uni-halle.de

Tospoviruses are now recorded from more than 500 plant species in over 50 families. These viruses use only about 11 thrips vectors to move from one plant to another. The transmission of the virus by adult thrips is only successful if the first or early second larval stage acquires the virus during feeding on infected plant tissue. Wijkamp I *et al*. 1996 (*Annals of Applied Biology* 129: 303–313) determined a median acquisition access period (AAP50) and pointed out that transmission ability increases with the length of the AAP on virus-infected material given to thrips larvae of *Frankliniella occidentalis*. On the other hand Sakurai T et al. 2002 (In: Marullo R, Mound LA, editors. *Thrips and Tospoviruses: Proceedings of the 7^th^International Symposium on Thysanoptera*, 51–57. Canberra: Australian National Insect Collection) describe intraspecific variation in transmission of TSWV from several populations of western flower thrips in Japan and pointed out that males transmit TSWV with higher efficiencies than females. Developmental studies of ontogenetic stages of thrips including their inner morphology let these results appear in a completely new light (Moritz G et al. 2004. *Virus Research* 100: 143–149). The so called short germ band in thrips shows a top-down-segmentation with a successive segment specification ((Tautz D. 2004. *Developmental Cell* 7: 301–312)). Shortly after katatrepsis the lobed salivary gland material appears behind the brain. In the first larval stage, the small time frame for virus acquisition is determined when the epithel cells of the mid gut and the salivary gland cells come in tight contact because the brain is displaced out of the small larval head by large cibarial muscles. Variable acquisition rates are the result of movements of the brain and the growing mid gut in size and length. An effective virus transfer cell ensemble exists only for a few hours. The reposition of the brain and the formation of the oscillatory flight muscles end the possibility to acquire tospoviruses definitely

# Research article: Thrips Identification: Classical, digital or molecular?

**DOI:** 10.1673/031.007.2849

**Published:** 2007-05-04

**Authors:** G Moritz, LA Mound, S Kumm

**Affiliations:** ^1^ Martin-Luther-University Halle-Wittenberg, Developmental Biology, Domplatz 4, 06108 Halle, Germany.; ^2^ CSIRO Entomology, P.O. Box 1700, Canberra, ACT 2601, Australia gerald.moritz@zoologie.uni-halle.de

Situation: In the last decades the importance of pest thrips has increased considerably worldwide. These species are usually highly adaptable, with a well developed resistance against certain insecticides and with a tendency to be vectors of fungi, bacteria and viruses. Some of them are invasive, and are notorious for causing extensive crop damage (Morse CG, Hoddle MS. 2007. *Annual Review of Entomology* 51: 67–89). Their identification is important to understand their biology and to empower integrated pest management strategies. However, it is questionable how effective current taxonomic methods and research are to adequately serve these problems and future scenarios. Classical Identification: The development of dichotomous keys is time consuming, expensive to print, of most use to professional taxonomists with good reference collections and libraries, and mostly focus on specific thrips taxa of local interest; their effective market niche is thus limited. In contrast, computing power is now inexpensive and available globally, and we should not ignore this availability (Cranston PS. 2005. *Systematic Entomology* 30: 1–3). One recent development that makes dichotomous keys more functional is the software *Phoenix* (For further information: http://www.lucidcentral.com) that involves scanning a printed key and then manipulating it electronically. Digital Identification: Several computerized identification and information systems, especially for pest thrips, are available (Moritz G *et al*. 2001. http://lucidcentral.com/keys/viewKeyDetails.aspx?id=218; 2004. http://lucidcentral.com/keys/viewKeyDetails.aspx?id=304. These systems provide fully illustrated and user-friendly means of recognising most of the thrips species that have been recorded as pests in various parts of the world, and moreover, the 2001 version provides information about all of the recognised families of these insects. These multivariate computer keys have important advantages and features and include a range of information on the host plants, distributions and body structure of these thrips. Furthermore, you can use expert routes combined with original computerized colour pictures, movie clips and the possibility to compare the identified species with other species of the key. Molecular Identification: The latest thrips key combines an illustrated visual key with molecular methods based on the ITS-RFLP technique and allows the identification of all ontogenetic stages including eggs. Limitations and problems occur only in species pairs of recent origin (Tautz D. 2003. *Trends in Ecology and Evolution* 18: 70–74), for example *Frankliniella bispinosa* and *F. tritici*.

# Research article: Evolution and genetic diversity among Thysanoptera

**DOI:** 10.1673/031.007.2850

**Published:** 2007-05-04

**Authors:** L Mound

**Affiliations:** CSIRO Entomology, Canberra, Australia. Laurence.Mound@csiro.au

Three aspects of thrips will be discussed: their classification, evolution and genetic diversity. The long-established classification of the insect Order Thysanoptera is morphology based but is not derived from modern phylogenetic concepts. The emphasis on structural autapomorphies results in 50% of the genera being monotypic, and the higher taxa remain poorly defined. Evolutionary studies on thrips using molecular methods are in their infancy, but are starting to yield interesting results that are at variance with morphology. Molecular methods are also being used to examine the genetic diversity within and between thrips species, and to produce new systems for routine identification of some taxa.

# Research article: Plants, thrips, *Tospoviruses* — the enigmatic triad

**DOI:** 10.1673/031.007.2851

**Published:** 2007-05-04

**Authors:** L Mound

**Affiliations:** CSIRO Entomology, Canberra, Australia. Laurence.Mound@csiro.au

This talk will examine the inter-relationships between the three elements involved in this crop protection triangle, emphasizing the dependence of tospoviruses on thrips despite less than 0.2% of the 5500 known thrips species being recorded as vectors. However, the members of each of the three elements are remarkably variable within and between populations, thus making investigations unusually complex. The inter-relationships between the three types of biologists involved in such studies will therefore be considered, and the effectiveness of their collaborative research efforts. Finally consideration will be given to how we can investigate collaboratively the origins and trajectory of this horticultural equivalent of the AIDS epidemic.

# Research article: A bunyaviral-type virus with unusual genome structure is associated with the ringspot disease of European mountain ash (*Sorbus aucuparia* L.)

**DOI:** 10.1673/031.007.2852

**Published:** 2007-05-04

**Authors:** HP Muehlbach, N Mielke, W Benthack, N Schlatermund

**Affiliations:** University of Hamburg, Biocentre Klein Flottbek and Botanical Garden, Ohnhorststrasse 18, D-22609 Hamburg, Germany. muehlbach@botanik.uni-hamburg.de

European mountain ash trees (*Sorbus aucuparia* L.) suffer from a widespread disease, which is characterized by ringspot and mottling symptoms on leaves and by a gradual decay in general. We could isolate double stranded RNA (dsRNA) from symptomatic tissue, which is indicative of viral infection. Cloning and sequencing of putative viral RNAs allowed the characterization of a new virus associated with the mountain ash disease (Benthack *et al*. 2005. *Archives of Virology* 150: 37–52.). Fractions of dsRNA were extracted by column chromatography. A pattern of four dsRNA bands of approximately 7 kb, 2.3 kb, 1.5 kb, and 1.3 kb, respectively, was found in leaf samples of symptomatic mountain ash trees from various sites in Germany. No dsRNA was detected in asymptomatic trees. By random primed reverse transcription, DOP-PCR (degenerate oligonucleotide primed PCR), cloning and sequencing, dsRNA-specific cDNA fragments were obtained. Using 5′-RACE analyses (rapid amplification of cDNA ends), modified by biotin labelling and magnetic separation, longer cDNAs could be enriched. With our cloning strategy a cDNA of 7.0 kb in length was obtained first. The corresponding RNA harbours one ORF with homology to the RNA dependent RNA polymerase (RdRP) of members of the family Bunyaviridae. It shows all conserved sequence motifs of the bunyaviral RdRP and also the typical terminus sequences at its 5′- and 3′ -end. Primers derived from the terminus sequences allowed the subsequent identification of three further RNAs of 2.3, 1.5 and 1.3 kb. The corresponding ORFs encode a putative glycoprotein precursor, a putative nucleocapsid protein, and a protein of unknown function. In situ hybridization studies using digoxigenin labelled riboprobes for the viral RNA 1 and RNA 3 showed a scattered pattern of virus accumulation in the mesophyll of mountain ash leaves. The dsRNA pattern, the sequence information and the present image of the viral genome organisation strongly indicate that a new plant RNA virus with some similarity to Bunyaviridae is associated with the mountain ash ringspot disease. (Benthack W et al. 2005)

# Research article: Environmental factors affecting *Tomato spotted wilt virus* (TSWV) symptom expression

**DOI:** 10.1673/031.007.2853

**Published:** 2007-05-04

**Authors:** S Mullis, A Csinos, R Gitaitis

**Affiliations:** Department of Plant Pathology, University of Georgia, P.O.Box 748, Tifton GA 31793 swmullis@uga.edu

Many environmental factors play a vital role in the expression of the *Tomato spotted wilt virus* (TSWV) disease. Temperature, rainfall, soil types, farmscape and thrips population density are factors that have been shown to have an impact on the expression of TSWV symptoms. While many plants can remain non-symptomatic, disease expression is the devastating aspect of TSWV infections. The degree and severity of the symptom expression is a complex set of events and factors that include the species of plant, plant age, plant condition, viral strain, and environmental conditions. Over a three year period, the timing of TSWV infections and symptom expression in tobacco plants were correlated with the overlying environmental factors affecting expression of the disease. Test plots were observed for a myriad of symptoms that ranged from single local lesions to plant desiccation due to TSWV. DAS-ELISA was used to determine the presence TSWV infection, and any symptoms that were present were documented for severity, type, and location. Rainfall, temperature, soil types, farmscape layout, and thrips density were evaluated for relation to disease expression. Although some of these factors are directly related to the ability of thrips to transmit TSWV to susceptible plants, this study concentrated on how these conditions affected TSWV disease expression.

# Research article: Complete sequence of the glycoprotein genes of four tospovirus species belonging to the American cluster

**DOI:** 10.1673/031.007.2854

**Published:** 2007-05-04

**Authors:** T Nagata, FA Lovato, KR Carvalho, RA Sodr, AK Inoue-Nagata, AC Ávila, RO Resende

**Affiliations:** ^1^ Ciências Genômicas e Biotecnologia, Universidade Católica de Brasilia, DF, Brazil.; ^2^ Ciências Biológicas, Universidade Católica de Brasília, DF, Brazil.; ^3^ Dept. Biol. Cel. Universidade Católica de Brasília, DF, Brazil..; ^4^ Empresa Brasileira de Pesquisa Agropecuária -Hortaliças. Brasília, DF, Brazil. rresende@unb.br

Several tospovirus species are often found in Brazil causing severe economic losses in horticultural and ornamental crops. Their N genes are fully sequenced, however, some important genes as the glycoprotein precursor (G) are still to be characterized. Due to its essential roles in the virus-vector interaction, the G gene of *Tomato chlorotic spot virus* (TCSV), *Groundnut ringspot virus* (GRSV) Zucchini lethal chlorosis virus (ZLCV) and Chrysanthemum stem necrosis virus (CSNV) were cloned and sequenced. Approximately 85% of the genomic segment of the M-RNA (4 Kbps) of all the four species was amplified by RT-PCR using specific and degenerated primers and cloned in the plasmid vector, pCR4 (Invitrogen). TCSV and GRSV shared 92% identity, while with Tomato spotted wilt virus (TSWV) identity were 81 and 79%, respectively. The G sequence of ZLCV had the highest identity of 76% with CSNV and 74% with TSWV, while CSNV was closely related to TSWV sharing 78% identity. Phylogenic analysis of the glycoprotein genes corroborated the close relationship among the tospoviruses belonging to the American cluster. The parameters on tospovirus taxonomic classification are discussed. Financial Support: CNPq (CBAB), Embrapa, UnB, UCB, FINATEC

# Research article: Epidemiology of *Tospoviruses* in South and Southeast Asia: Current status and future prospects

**DOI:** 10.1673/031.007.2855

**Published:** 2007-05-04

**Authors:** RA Naidu, S Adkins, KS Ravi, P Chiemsombat, RK Jain, HS Savithri, O Gajanandana, V Muniyappa, DJ Riley

**Affiliations:** ^1^ Washington State University-IAREC, Prosser, WA, 99350, USA.; ^2^ USDA-ARS-USHRL, Fort Pierce, FL 34945, USA; ^3^ Mahyco Research Center, Jalna 431203, India; ^4^ Kasetsart University, Kamphaeng Saen Campus, Nakorn Pathom 73140, Thailand;; ^5^ Indian Agricultural Research Institute, New Delhi 110012, India; ^6^ Indian Institute of Science, Bangalore 560 024, India; ^7^ National Center for Genetic Engineering and Biotechnology, Pathumthani 12120, Thailand; ^8^ University of Agricultural Sciences, Hebbal Campus, Bangalore 560 024, India; ^9^ The University of Georgia, Coastal Plain Experiment Station, Tifton, GA 31793, USA. naidu@wsu.edu

At least seven of the fourteen established tospoviruses occur in South and Southeast Asia (S&SEA). *Peanut bud necrosis virus* (PBNV), *Peanut yellow spot virus* (PYSV) and *Watermelon bud necrosis virus* (WBNV) were reported from India. Among them, PBNV and WBNV belong to serogroup IV and PYSV belong to serogroup V. The occurrence of *Iris yellow spot virus* (IYSV) has recently been reported in onion for the first time from India. Studies on the epidemiology of PBNV have indicated that the virus has a broad geographic range and infects several economically important crops in the family Leguminoceae and Solanaceae. In addition, other crops like cotton and sesame, and several weed species were found to be naturally infected with PBNV. Serology and comparative analysis of nucleocapsid gene sequence of PBNV isolates from different host plants revealed that they are closely related. The host range of WBNV appears to be restricted to members of the family Cucurbitaceae. The host range of PYSV is not yet determined. PBNV and WBNV were reported to be vectored by *Thrips palmi. Scritothrips dorsalis* was shown to be the principal vector of PYSV. The presence of *Thrips tabaci* in India, the main vector of IYSV infecting onion in other countries, is yet to be known.

PBNV, *Watermelon silver mottle virus* (WSMV), *Melon yellow spot virus* and isolates related to *Capsicum chlorosis virus* (CaCV), all belonging to serogroup IV, have been documented in Thailand. Recent studies, based on serology and nucleotide sequence analyses of the nucleocapsid gene, have shown that WSMV and MYSV and tospovirus isolates related to CaCV infect several economically important crops in the family Solanaceae and Leguminoceae. Besides PBNV, tospovirus isolates related to CaCV were found in peanuts. Although *T. palmi* was observed predominantly in cucurbits and *S. dorsalis* was found to be dominant in peanuts, their ability to transmit these tospoviruses has not been studied. Recently, *Ceratothrips claratris* has been reported as a competent vector of a tospovirus closely related to CaCV. *Frankliniella occidentalis* (Western Flower Thrips), the principal vector of many tospoviruses in different countries around the world, has not yet been recorded in S&SEA. The occurrence of IYSV in India and isolates of CaCV in Thailand may suggest the expansion of tospoviruses to new geographic regions, perhaps due to changes in agricultural practices and shifting cropping patterns and international trade and commerce. Consequently, improved knowledge of tospovirus diversity and tospovirus-vector thrips species interactions is a necessary precursor for the deployment of comprehensive integrated disease management strategies to mitigate losses caused by different tospoviruses in S&SEA.

# Research article: Life history traits and nucleotide sequences of mt-DNA of thelytokous and arrhenotokous races of *Thrips nigropilosus* Uzel

**DOI:** 10.1673/031.007.2856

**Published:** 2007-05-04

**Authors:** S Nakao, M Muraji

**Affiliations:** ^1^ Lab. of Landscape Entomology, Faculty of Systems Engineering, Wakayama University, Sakaedani, Wakayama 640-8510, Japan.; ^2^ Lab. of Insect Molecular Evolution, National Institute of Agrobiological Sciences, Tsukuba, Ibaraki 305-8634, Japan nakao@sys.wakayama-u.ac.jp

*Thrips nigropilosus* is a pest of chrysanthemum in Japan, and the females exhibit wing polymorphism. It is known that mode of reproduction and critical daylengths for induction of reproductive diapause and brachypter production in females show geographical variations (Nakao S *et al*. 1997. *Environmental Systems Research* 25: 665–669; Nakao S, Yabu S. 1998. *Japanese Journal of Entomology* 1: 9–19). Prevalent mode of reproduction of the wild populations of *T. nigropilosus* was examined in Wakkanai city, Hokkaido Island, Japan. Furthermore, photothermal responses, reproductive schedule, fecundity, and partial nucleotide sequences of mitochondrial DNA were compared between thelytokous and arrhenotokous races. Percentages of thelytokous thrips were 0.8–4.0% in the wild population. Significant differences between thelytokous and arrhenotokous races were not observed in the duration of pre-ovarial maturation and oviposition periods at 18C and 15L-9D condition. The mean numbers of eggs deposited by thelytokous and arrhenotokous females were ca.63 and ca.142 at the same condition, respectively, and the hatchabilites were not different from each other. At 18C, the critical daylength for the production of the brachypters and the induction of the reproductive diapause of thelytokous females was 14–14.5h, and that of arrhenotokous ones was 13–14h. Even under short-daylength, the higher temperatures inhibited the development of the brachypters and the induction of the diapause in thelytokous and arrhenotokous females. The molecular phylogenetic tree of the CO I nucleotide sequences revealed that *T. nigropilosus* are divided into two clusters which consist with the thelytokous race and the arrhenotokous ones. This result was consistent with their esterase zymograms (Nakao S, Yabu S. 1998. *Japanese Journal of Applied Entomology and Zoology* 42: 77–83). A previous study (Nakao S, Yabu S. 1998. *Japanese Journal of Entomology* 1: 9–19) had revealed that the arrhenotokous population of Wakkanai city has a dominant short-wing gene inducing brachypters in females irrespective of day length and temperature. Thus, the thelytokous race seems to be derived from an arrhenotokous female lacking the dominant gene, in the colder climatic periods or at a little north from the northern edge of Hokkaido Island.

# Research article: Reproductive biology of *Thrips tabaci* from New York onion fields

**DOI:** 10.1673/031.007.2857

**Published:** 2007-05-04

**Authors:** BA Nault

**Affiliations:** Department of Entomology, Cornell University, New York State Agricultural Experiment Station, Geneva, NY 14456 USA. ban6@cornell.edu

Knowledge of a pest's reproductive biology is important for developing effective management strategies. Onion thrips, *Thrips tabaci* Lindeman, is a serious pest of several field and vegetable crops and damaging populations can escalate quickly if not controlled. *Thrips tabaci* can reproduce sexually, but the most common mode of reproduction is thelytoky, a type of parthenogenesis in which unfertilized eggs develop into females. However, *T. tabaci* populations have been reported to reproduce via arrhenotoky (Kendall DM, Capinera JL. 1990. *Southwestern Entomologist* 15: 80–88), a type of parthenogenesis in which unfertilized eggs develop into males. Deuterotoky, a third type of parthenogenesis characterized by unfertilized eggs developing into either males or females, has been documented in the thrips species, *Apterothrips apteris* (Mound L. 1992. In: Cameron EA, et al., editors. *Proceedings of the 1991 Conference on Thrips (Thysanoptera): Insect and Disease Considerations in Sugar Maple Management*, 2–14), but not in *T. tabaci*. Male onion thrips are frequently encountered in New York and Ontario onion fields (MacIntyre-Allen JK. 2004. *Population dynamics*, *insecticide resistance and management of onion thrips*, *Thrips tabaci Lindeman*, *in southwestern Ontario*. Ph.D. dissertation, University of Guelph, Guelph, Ontario). Although arrhenotoky is assumed to be the principal mode of reproduction in these populations, it has not been empirically differentiated from deuterotoky. To identify the mode or modes of reproduction by *T. tabaci* populations in New York, the progeny of virgin females from these fields had to be identified. In 2002–2004, progeny of virgin females originating from 23 commercial onion fields were identified. About half of the populations were strictly thelytokous (n=13) whereas the other half included a combination of thelytokous, arrhenotokous and deuterotokous individuals (n=10). The factor(s) responsible for triggering the production of males is not known, but has been hypothesized to occur when temperature increases (Moritz G. 1997. In: Lewis T, editor. *Thrips* *as* *Crop Pests*, 2: 15–63. CAB International, Cambridge: University Press) or when populations develop resistance to insecticides (Kendall and Capinera 1990). Neither hypothesis has been examined. In 2002–2003, the level of susceptibility of *T. tabaci* populations to several commonly used insecticides was characterized using a laboratory bioassay (Shelton AM *et al*. 2003. *Journal of Economic Entomology* 96: 1843–1848). Concurrently, the modes of reproduction by the *T. tabaci* populations were identified and a correlation between resistance and reproductive mode examined. There was no relationship between susceptibility to insecticides and a particular mode of reproduction. The effect of temperature on production of males also was examined. A single population from western New York was separated into three sub-populations, each of which was maintained at 85°, 75° or 65° F for three consecutive generations. Only females were produced, indicating that high temperature alone is not responsible for production of males. Other factors that may affect the mode of reproduction in *Thrips tabaci* populations are discussed.

# Research article: Genetic structure of TSWV isolates collected from epidemics in North Carolina (2002) and California (2003)

**DOI:** 10.1673/031.007.2858

**Published:** 2007-05-04

**Authors:** SL New, JA Abad, I Carbone, GG Kennedy, M Tsompana, JW Moyer

**Affiliations:** ^1^ Department of Plant Pathology, North Carolina State University, Raleigh, NC.; ^2^ Center for Integrated Fungal Research, Department of Plant Pathology, North Carolina State University, Raleigh, NC; ^3^ Department of Entomology, North Carolina State University, Raleigh, NC; ^4^ Department of Biology, University of North Carolina-Chapel Hill, NC. james_moyer@ncsu.edu

*Tomato spotted wilt virus* (TSWV) has been known to cause wide spread epidemics in a number of crops with increased frequency. In addition, there is constant geographic expansion of the virus. In this study, we compare and characterize the genetic structure of infected isolates obtained in epidemics of North Carolina and California. For this study, sequence of the entire nucleocapsid (N) gene and the glycoprotein precursor (GnGc) in addition to partial fragments of the non-structural protein (NsM) and the RNA-dependent-RNA-polymerase (RdRp) were generated from 15 isolates from North Carolina and seven isolates from California. Preliminary analysis for all coding regions sequenced in this study revealed the clustering of all isolates from North Carolina with previously sequenced isolates collected from the Southeastern region of the United States. California isolates clustered in at least two distinct groups. Statistical tests were used to evaluate the genetic differentiation between isolates based on host or geographic origin. The null hypothesis of no differentiation between subgroups based on host was not rejected. However, the differentiation of geographical subpopulations was supported statistically at all loci and in the analysis of the combined sequence. Additionally, synonymous changes specific to geographical subpopulations were determined from the amino acid translations. Further analysis was used to elucidate the evolutionary properties of the different geographical subpopulations.

# Research article: Color morphs of western flower thrips in California: Phenotypic or genetic variation?

**DOI:** 10.1673/031.007.2859

**Published:** 2007-05-04

**Authors:** C O'Donnell

**Affiliations:** University of California Davis, One Shields Avenue Davis California 95616, USA caodonnell@ucdavis.edu

Western flower thrips, *Frankliniella occidentalis* (Pergande), is a well known pest in California and is polymorphic for color, with populations varying from nearly black to pale yellow. This type of color polymorphism has been noted throughout the global distribution of this insect. Several hypotheses have been proposed for why these color morphs occur, i.e. variation in temperature or host plant, or genetic differences within the species. We have compared sequence data from the nuclear gene ITS and the mitochondrial genes COI and 16S to analyze genetic variation among western flower thrips from many regions of California, as well as selected populations from Europe, Asia, India and several states in the USA. A dendrogram revealing the relationship between color polymorphism and genetic similarity in western flower thrips will be presented.

# Research article: Phylogenetic Analysis of the Genera *Frankliniella* and *Thrips* (Thysanoptera: Thripidae): the Evolution of Virus Vectors

**DOI:** 10.1673/031.007.2860

**Published:** 2007-05-04

**Authors:** C O'Donnell

**Affiliations:** University of California Davis, One Shields Avenue Davis California 95616, USA. caodonnell@ucdavis.edu

The genera *Thrips* and *Frankliniella* comprise 460 species from among the 2300 species within the Thripidae (2300 spp.) and ten of these species transmit topsoviruses. It is not known why only these few species serve as virus vectors or whether the vector species within each genus are related. We aim to provide a phylogeny that better explains the species relationships within *Thrips* and *Frankliniella*. In this project our specific objectives are to determine if the vector species within each genus are related. The phylogenetic analysis includes nucleotide sequence data from two mitochondrial genes (16S and COI) and two nuclear genes (18S and 28S) from representative species of *Thrips*, *Frankliniella* and other genera of Thripidae with outgroups from Aeolothripidae, and Phlaeothripidae families.

# Research article: Management of *Tomato spotted wilt* and Its Impact on Tomato Production

**DOI:** 10.1673/031.007.2861

**Published:** 2007-05-04

**Authors:** SM Olson, T Momol, J Funderburk

**Affiliations:** North Florida Research and Education Center, IFAS, University of Florida, Quincy, FL. smolson@ifas.ufl.edu

Spotted wilt caused by *Tomato spotted wilt virus* (TSWV) and vectored mainly by western flower thrips (*Frankliniella occidentalis*) and tobacco thrips (*F*. *fusca*) is a major disease of tomato in southeastern U.S. Research has shown that primary infection can not be controlled by insecticide treatments, but secondary can be controlled. Field experiments were conducted to determine the separate and integrated effects of UV-reflective mulch (UVRM), acibenzolar-S-methyl (Actigard) and insecticides to manage TSW in tomato. Highly UV-reflective and black mulch covered plots were treated with Actigard and insecticides in field experiments. TSW incidence was determined from late April to June. Disease incidence was reduced significantly in highly UVRM and Actigard treated plots. The highly UVRM alone was effective in reducing disease incidence every year tested. Actigard significantly reduced spotted wilt incidence in two years when TSWV infection pressure was high. Highly UVRM and Actigard were effective on reducing the primary spread of spotted wilt in field-grown fresh market tomatoes. The combination of highly UVRM, Actigard, and insecticides was the most effective integrated treatment and reduced tomato spotted wilt by as much as 81%. Information will be presented on the effect of metalized mulches and Actigard on production of tomatoes and peppers. Use of the metalized mulches has never resulted in a reduction in yield on a per hectare basis but at times yield has been reduced on a per plant basis due to reduced soil temperatures under the metalized mulch. Use of Actigard has not resulted in reduction of tomato yields. One drawback of the metalized mulch is that it is more expensive than traditional black mulch ($940/ha versus $380/ha).

# Research article: Epidemiological and molecular aspects of *Iris yellow spot virus* in the Pacific northwest

**DOI:** 10.1673/031.007.2862

**Published:** 2007-05-04

**Authors:** HR Pappu, L duToit, H Schwartz

**Affiliations:** ^1^ Department of Plant Pathology, PO Box 646430, Washington State University, Pullman, WA; ^2^ WSU - Northwestern Washington Research & Extension Center, Mount Vernon, WA; ^3^ Colorado State University, Department of Bioagricultural Sciences and Pest Management, Fort Collins, CO. hrp@wsu.edu

*Iris* *yellow spot virus* (IYSV), of genus *Tospovirus*, family *Bunyaviridae*, has been endemic in onion crops in the Treasure Valley of Idaho for over a decade (Hall JM *et al*. 1993. *Plant Disease* 77: 952), where the virus was largely confined to seed crops. Epidemics of IYSV were observed in bulb crops beginning in 2000, and the virus has since been detected in Arizona, California, Colorado, New Mexico, Oregon, Utah and Washington in the western US, and in Georgia in the southeastern US. In Washington, IYSV was first observed in one county with a few symptomatic plants in 2002. By 2004, the virus was detected in all major counties for onion production, with some fields showing ?90% incidence of symptomatic plants. Severe outbreaks in seed crops in central Oregon (Crowe F, Pappu HR. 2004. *Plant Disease* 89:105) and seed and bulb crops in Washington during 2004 suggest favorable conditions for the virus and vector buildup. The rapid spread of the virus and its emergence as a serious disease constraint raises several questions about the epidemiology of the disease and the factors that contributed to the shift towards increased incidence. Rapid spread and the increased impact of IYSV in northwest maybe attributable to: 1) prevalence of the vector, *Thrips tabaci*, 2) the ‘green bridge’ effect from overlapping biennial seed and annual bulb crops, and 3) limited resistance in commercial cultivars. Nucleoprotein (NP) gene sequences of several isolates from the Pacific Northwest of the US were determined. Onion samples showing symptoms indicative of IYSV infection were collected from commercial seed and bulb crops in Colorado, Idaho, and Washington in 2003 and 2004. Using a RT-PCR assay, the NP gene was amplified and cloned from selected isolates. Dendrograms based on the translated amino acid sequences of the NP gene showed that isolates from Colorado, Idaho, and Washington were highly similar to one another and formed a tight cluster and shared a high degree of sequence identity with the Lisianthus isolate from Japan. Isolates from several counties within the Washington state were closer to each other and with the Idaho isolate. Isolates from Australia, Brazil, Israel, Japan (*Alstroemeria* isolate), the Netherlands, and Slovenia diverged from the isolates sequenced in this study. ELISA, PCR, and real-time PCR assays are being developed for improved detection of IYSV. Management options that are being explored include vector management, and use of SAR-inducers such as acibenzolar-S-methyl.

# Research article: The development of a comprehensive IPM program to manage western flower thrips and *Tospoviruses* in a bedding plant nursery in California

**DOI:** 10.1673/031.007.2863

**Published:** 2007-05-04

**Authors:** M Parrella, T Costamagna

**Affiliations:** Department of Entomology, University of California, Davis, CA. USA. mpparrella@ucdavis.edu

Thrips and tospovirus [most notably the western flower thrips (*Frankliniella occidentalis*) and *Impatiens necrotic spot virus*, are perennial problems for most commercial floriculture operations in California. With large thrips' populations developing in areas adjacent to greenhouse/nurseries, migration of viruliferous thrips into sensitive cropping areas occurs every year. Unless a comprehensive management plan is in place, growers are often forced into a reactionary mode, where the only recourse is the use of regular pesticide sprays. Depending on the degree of migration, possible resistance in the target populations and the selection and type of application of pesticides, pesticides alone are often not be enough to provide satisfactory thrips and virus control. This past year we have worked with one large nursery where they have sprayed as much as 35 times per year for control of thrips (virus) and other pests, with little effect. We have dramatically reduced the number of sprays used in this greenhouse/nursery and have achieved much better control of thrips and virus. We hope that the steps taken in this operation could serve as a model for other nurseries to follow. Cultural/physical tactics adopted by this greenhouse/nursery included adding concrete floors to some of the greenhouses, sanitizing greenhouses between crops, using thrips-proof screening, treating plants prior to moving them from the field to the greenhouse, adding baffles and/or screened foyers at the entrance to the greenhouses, and planning crop location and movement to minimize contamination with thrips. A monitoring program was put into place for thrips and tospovirus, predatory mites were included as an overall control tactic, and pesticide selection was based on bioassays conducted with thrips collected from this nursery. Many of these tactics are not minor and required considerable expense by the nursery. The overall result has been better pest control with fewer pesticides.

# Research article: Developing *Capsicum* and tomato cultivars with resistance to *Tospoviruses* in Australia

**DOI:** 10.1673/031.007.2864

**Published:** 2007-05-04

**Authors:** DM Persley, M Sharman, D McGrath, S Garland

**Affiliations:** ^1^ Cooperative Research Centre for Tropical Plant Protection, St Lucia, Australia 4072.; ^2^ Department of Primary Industries and Fisheries, Bowen, Australia 4805 denis.persley@dpi.qld.gov.au

*Capsicum* (pepper) crops in Queensland, Australia can be seriously affected by both *Tomato spotted wilt virus* (TSWV) and *Capsicum* chlorosis virus (CaCV). The latter virus, first described from Australia, is a member of the serogroup IV or *Watermelon silver mottle virus* serogroup (McMichael *et al*. 2002). Although useful resistance to TSWV has not been found in *Capsicum annuum*, resistance expressed as a hypersensitive response, and controlled by the dominant gene *Tsw*, has been found in several *C*. *chinense* accessions, including PI 152225 and PI 159236, and introgressed into *C. annuum* lines (Roggero P et al. 2001. Proceedings of the 7^th^ International Symposium on Thysanoptera, University of Reggio Calabria, Italy). The *Tsw* gene is not effective against CaCV (McMichael L *et al*. 2002. *Australasian Plant Pathology* 31:231–239). When 38 *C*. *chinense* accessions from the USDA/University of Georgia collection were screened by sap inoculation with CaCV, one line (PI 290972) developed a hypersensitive response on inoculated leaves with premature leaf abscission and no detectable virus by ELISA and RT-PCR assays of new growth leaves. Six sub-lines of PI 290972 were established by self pollination; all were resistant to CaCV and one was also resistant to TSWV, developing a hypersensitive response following inoculation. Resistance to both CaCV and TSWV was dominant in the F1 generation of crosses between PI 290972 and two bell capsicum lines. This material has now been advanced to a third backcross. Selection efficiency has been enhanced by using a dominant PCR—based molecular marker to select segregates for CaCV resistance. Results were confirmed by manual inoculation of backcross populations which supported a single dominant gene model for inheritance of the resistance. The *Sw-5* gene for TSWV resistance in tomato has been incorporated into elite tropically-adapted lines by backcrossing. Selection efficiency has been improved by the development of a PCR-based marker system that consists of coamplification of a dominant marker representing the *Sw-5* gene sequence and a modified cleaved amplified polymorphic sequence (CAPS) marker as a positive control and indicator of genotype (Garland S *et al*. 2005. *Australian Journal of Agricultural Research* 56: 285–289).

# Research article: Impeded spread of *Tomato spotted wilt virus* to pepper plants less preferred by *Frankliniella occidentalis*

**DOI:** 10.1673/031.007.2865

**Published:** 2007-05-04

**Authors:** D Peters, P Maris, N N Joosten, RW Goldbach

**Affiliations:** Laboratory of Virology, Wageningen Agricultural University, Binnenhaven 11, 6709 PD Wageningen, the Netherlands. dick.peters@wur.nl

Among its many hosts, resistance to Tomato spotted wilt virus (TSWV) has been incorporated in a limited number of plant species, like groundnut, pepper and tomato. This resistance occurring in natural sources has been overcome by some TSWV isolates in pepper and tomato.. Artificial resistance has also been obtained by insertion of the N gene of TSWV or parts of this gene in some hosts, but has not widely been applied in the field. Not much attention has thus far been spent on the significance of resistance to the vector to control spread of TSWV. This may be due to the cumbersome screening techniques to be used and the likelihood that this resistance is polygenic. In addition, this resistance may have either antagonistic or synergistic effects on the spread of tospoviruses. In the former case this resistance may promote the spread of the virus when incoming thrips may search for new hosts after probing non-preferred hosts. In the latter case, the spread of the virus might become restricted when thrips do not migrate from a non-preferred host. To get answers on these questions the spread of TSWV among thrips-susceptible and thrips-resistant pepper plants, which were equally susceptible to TSWV, was studied. All plants of the accession Pikante Reuzen (susceptible to thrips) exposed to infectious adult *Frankliniella occidentalis* thrips became infected in non-choice tests, compared to only 40% of the plants of the more resistant accession CPRO-1. Infection of the resistant plants could be attributed to inoculation by the released thrips, but not to a secondary spread as the reproduction of thrips and larval survival was strongly impeded on these plants. More thrips-susceptible than -resistant plants became also infected when the thrips were released in a mixed stand. All resistant plants became finally infected probably due to secondary spread of virus from the infected susceptible plants. Further studies revealed that the behavior of thrips did not differ on leaves of the susceptible and resistant plants, although the thrips dispersed at a slightly higher rate from resistant plants. Thrips populations grew considerably faster on susceptible plants when they were released on resistant plants in a mixed stand, whereas only a few specimens were detected on resistant plants when released on susceptible plants. The results show evidently that resistance to thrips is a proper trait to impede the spread of tospoviruses as both primary and secondary infections become restricted due to a lower infection success, a lower acceptance rate of the plant by thrips and a decreased population development. (Maris PC et al. 2003. Phytopathology 93: 96–101; Maris PC et al. 2003. Phytopathology 93: 1223–1227; Maris PC et al. 2004. Entomologia Experimentalis et Applicata 113: 149–155).

# Research article: Diversity, similarity and seasonal variation of the thysanopterofauna in Itapuã State Park, Viamão, RS, Brazil

**DOI:** 10.1673/031.007.2866

**Published:** 2007-05-04

**Authors:** SJM Pinent, CE da C Pinent, HP Romanowski, LR Redaelli, A Cavalleri

**Affiliations:** ^1^ Depto. Fitossanidade - UFRGS, Porto Alegre, RS.; ^2^ Depto. Entomologia - EMBRAPA UVA E VINHO, Bento Gonçalves, RS; ^3^ Dep. Estatística - PUCRS, Porto Alegre, RS silviapi@portoweb.com.br

The diversity, similarity and seasonal variation of the thysanopterofauna in different microhabitats were studied in the “Parque Estadual de Itapuã” (30°22′S 51°02′W), RS, southern Brazil. Between June 1999 and May 2001, shoots (n = 1,274), flowers (n = 774), grass tussocks (n = 596) and leaf litter (n = 603) were sampled systematically in 20 points on four trails (T1- Pedreira beach, T2- Araçá beach, T3- Lagoinha, and T4- Grota hill). This resulted in 2,197 adult thrips determined in 73 species. The seasonal variation points to both N and S higher during summer and lower in winter. Estimated values for diversity were DMg = 9.357 and H' = 2.802. Dominance was low overall in the sample (1-D = 0.893). The highest abundance was obtained for flowers (N = 1,224). Although less abundant in individuals (N = 158), the leaf litter was rich in species. T2 had comparatively high numbers of thrips, resulting also in a high number of species (S = 43). The fauna showed very low similarities among microhabitats (MH lower than 0.2); among trails, similarity was higher: MH = 0.83 between T2 and T4. T1 had the most distinct fauna. We discuss the importance of sampling different types of vegetation and microhabitats so that specialised and generalist species can be recorded. (N = abundance; S = species richness; D = Simpson's index; 1 — D = reciprocal Simpson index; DMg = Margaleff index; H' = Shannon-Wiener index; MH = Morisita-Horn's similarity index).

# Research article: Thrips species (Thysanoptera) on strawberry, persimmon and grape in the Gaúcho Highlands, Rio Grande do Sul State, southern Brazil

**DOI:** 10.1673/031.007.2867

**Published:** 2007-05-04

**Authors:** SMJ Pinent, CE da C Pinent, M Button, LR Redaelli

**Affiliations:** ^1^ Departmento Fitossanidade, Universidade Fed Rio Grande Sul, Porto Alegre, RS, Brazil.; ^2^ Departmento Entomologia, Empresa Brasileira de Pesquisa Agropecuária Uva e Vinho, Bento Gonçalves, RS, Brazil.; ^3^ Departmento Estatística, Pontificia Universidade Catolica do Rio Grande do Sul, Porto Alegre, RS, Brazil. silviapi@portoweb.com.br

A survey of thysanopterans occurring on persimmon, grape and strawberry in orchards of the Gaúcho Highlands region (Serra Gaúcha) (24°09′S 51°31′W), north-western Rio Grande do Sul, southern Brazil, was carried out to study thrips incidence, specially pest species. Seasonal samples were collected on persimmon and grape, and monthly from September/2004 to February/2005 on strawberry, which showed higher incidence of thrips. In each orchard, 10 plants, flowers, shoots and fruits were manually collected on a randomised way. In the lab, thrips were separated from the plants, preserved in AGA solution, mounted on microscopy slides and identified. From a total of 794 individuals, 365 adults and 429 immatures, ten thrips species were identified. From those, only the *Frankliniella occidentalis* (n = 45) occurred on the three crops, showing a higher degree of polyphagy and causing damage on grape. *Frankliniella schultzei* was the most abundant (n = 147) and was found causing damage by feed only on strawberry, followed by *Frankliniella gemina* (n = 106). Of the species considered as crop pests we found *F. occidentalis*, *F. schultzei* and *Thrips tabaci* (n = 9) on strawberry and *Heliothrips haemorrhoidalis* (n = 5) on grape. However, according to Mound (2005), *T. tabaci* has been losing this condition and some of the damage attributed to thrips by the farmers may be caused by other insects as aphids or mites and also by a deficit of bee pollination or virus incidence. Overall 75,3% occurred inside the flowers, being 16,2% on leaves and 8,5% on fruits. On fruit, they were observed on the bottom, under the sepals, and on leaves under the surface. The knowledge of the species associated to different crops is fundamental for the implementation of integrated pest management with emphasis on natural biological control. (Mound LA. 2005. *Annual Review of Entomology* 50: 247–69).

# Research article: Epidemiology of *Tomato spotted wilt virus* (TSWV) in vegetables relative to thrips population dynamics

**DOI:** 10.1673/031.007.2868

**Published:** 2007-05-04

**Authors:** D Riley

**Affiliations:** Entomology Department, University of Georgia, P.O. Box 748, Tifton, Georgia USA. dgr@uga.edu

The epidemiology of thrips-vectored plant disease includes the study of the causes of the disease, analyzing disease occurrence and distribution, and understanding the factors that govern it's spread. The ultimate goal of epidemiological studies is to control disease in host crop populations. For thrips- vectored Tospovirus, epidemics require the interaction of the thrips vector, the pathogen host plant and the pathogen. In Georgia, the temporal and spatial dynamics of Tomato spotted wilt virus (TSWV) in Solanaceous vegetable crops, such as tomato, appear to be related to the dynamics of the two main thrips vectors species, *Frankliniella fusca* (Pergande) and the flower thrips, *Frankliniella occidentalis* (Hinds). The dynamics of thrips vectors in weed hosts during the winter have been reported to be associated with disease incidence in the Spring cropping season. In studies where weed host of TSWV and thrips were maintained adjacent to a tomato field, elevated levels of TSWV were observed in the tomato field compared to fields without the weed source for TSWV. The dominant thrips vector species in weed host studies in the early Spring appear to be *F. fusca*. Also, early colonizers of the foliage of tomato plant were observed to be predominantly *F. fusca* prior to flower formation. Since early inoculation of TSWV results in severe yield reduction, yield loss correlates well with *F. fusca* populations. In most studies, *F*. *occidentalis* was the dominant TSWV vector species on the plant once flowers are present on the crop. In addition, late season incidence of TSWV is better correlated with the incidence of *F. occidentalis* than *F*. *fusca*, suggesting that *F. occidentalis* is the more effective vector later in the tomato growing season. *Frankliniella occidentalis* has also been shown to be more positively affected by pollen deposition on leaf tissue in terms of settling and oviposition than *F*. *fusca*. We suspect that ambient pollen dynamics in the Southeastern USA directly affects population dynamics of flower thrips and indirectly affects the epidemiology of TSWV. Another factor that could be important in the epidemiology of TSWV is thrips feeding preference. Studies suggest that *F*. *fusca* prefers young tomato plant foliage compared to older leaf tissue and avoids imidacloprid- treated leaf tissue. On the other hand, *F*. *occidentalis* is less affected by tomato plant age and imidacloprid treatment tends to enhance feeding. Also, imidacloprid treatment and pollen deposition in peanut enhances settling by *F*. *occidentalis* on leaf tissue. A two-vector model is proposed to explain some of the observed trends in TSWV epidemiology in the Georgia.

# Research article: Evaluation of hot pink traps to monitor pests on ornamental crops

**DOI:** 10.1673/031.007.2869

**Published:** 2007-05-04

**Authors:** KL Robb, J Newman, HS Costa, S Parker

**Affiliations:** ^1^ University of California Cooperative Extension, 5009 Fairgrounds Rd, Mariposa, California, USA; ^2^ University of California Cooperative Extension, Counties of Ventura and Santa Barbara, USA; ^3^ Department of Entomology, University of California, Riverside, California, USA.; ^4^ University of California Cooperative Extension, County of San Diego, California, USA heather.costa@ucr.edu

Industry standards for ornamental crops are blue or yellow sticky traps for thrips (Heinz *et al*. 1992, *Journal of Economic Entomology* 85:2263–2269). Tsuchiya *et al*. (1995, *Japanese Journal of Applied Entomology and Zoology* 39:313–319) demonstrated a significant preference by western flower thrips for hot pink in mandarin oranges. If pink proved significantly more attractive to western flower thrips in flower crops, we would consider changing the color of the petunia tospovirus indicator plant stands (Robb *et al*. 1999. http://comserv.ucdavis.edu/CESanDiego) to pink to increase the attractancy of the indicator plants. We were also interested in determining whether there would be an advantage to switching from yellow sticky traps to pink. Trials were conducted to verify color preference by western flower thrips and other insects in San Diego County and Ventura County. Blue, yellow and hot pink traps were created and placed in a field grown cut chrysanthemum crop. In each trial, 10 traps of each color were evaluated in a randomized design. The trial was replicated five times over 5 weeks. Greenhouse trials were conducted amongst a crop of impatiens bedding plants that were just beginning to show color. Although western flower thrips were the target pest, numbers of leafminers, whiteflies, aphids and fungus gnats captured were also recorded. All data were analyzed using ANOVA. Hot pink was not preferred by western flower thrips over blue. The number of western flower thrips on hot pink was not statistically different than the number caught on yellow sticky traps. Moreover, pink traps were no more attractive than yellow to aphids, leafminers, whiteflies or fungus gnats. As a result of these findings, we do not recommend changing the color of the petunia indicator plant stands used for detecting tospovirus infective thrips from blue to pink. There is also no advantage to using hot pink sticky traps for the other ornamental plant insects monitored in this trial, so no modifications to existing trap strategies are recommended.

# Research article: Population dynamics of the New Zealand flower thrips (*Thrips obscuratus*) and possible consequences for the incidence of Botrytis bunch rot in grapes

**DOI:** 10.1673/031.007.2870

**Published:** 2007-05-04

**Authors:** K Schmidt, SD Wratten, DAJ Teulon, MV Jaspers, RE Beever

**Affiliations:** ^1^ National Centre for Advanced Bio-Protection Technologies, PO Box 84, Lincoln University, Canterbury, New Zealand.; ^2^ New Zealand Institute for Crop & Food Research Limited, Private Bag 4704, Christchurch, New Zealand; ^3^ Landcare Research, Private Bag 912170, Auckland, New Zealand schmidtk@lincoln.ac.nz

*Thrips obscuratus*, the New Zealand Flower Thrips (NZFT) is endemic to New Zealand and is the most widespread of the New Zealand thrips. It is a well known pest of stonefruit, where its feeding activities cause distortion and damage to ripe fruit and act as a vector for *Monilinia fructicola*. This thrips species also occurs in vineyards during flowering, when adults and larvae feed on nectar and pollen, causing considerable loss of viable pollen and also damage the ovaries. *T. obscuratus* can achieve densities of up to two million per hectare in vineyards. Since it is so abundant, this species may have a major effect on grape and disease development. In the 2004/2005 season, different types of traps were used to monitor *T. obscuratus* flight activity and its presence at two field sites in the New Zealand South Island regions of Nelson and Canterbury. Initial results showed that large populations of airborne thrips at flowering probably originated from sources outside the vineyards. Their colonization of the vines led to high numbers of eggs and larval stages on bunches. The interaction between NZFT and Botrytis bunch rot was investigated in greenhouse and field trials by introducing a marker strain of *B. cinerea*, with and without NZFT, to grape flowers enclosed in fine silk bags. When NZFT was present on flowers, the berries developed corky scars and had a greater incidence of *B. cinerea* infection in some treatments.

# Research article: Tospoviruses Infecting Capsicum and Tomato in Australia

**DOI:** 10.1673/031.007.2871

**Published:** 2007-05-04

**Authors:** M Sharman, D Persley, L McMichael, J Thomas

**Affiliations:** ^1^ Cooperative Research Center for Tropical Plant Protection, St Lucia, Queensland, Australia 4072.; ^2^ Department of Primary Industries, Plant Pathology Group, 80 Meiers Road, Indooroopilly Australia 4068 murray.sharman@dpi.qld.gov.au

Tomato spotted wilt virus (TSWV) has caused serious sporadic epidemics in capsicum and tomato crops in all major growing regions of Australia since it was first described in the 1920s. The frequency and distribution of outbreaks increased in the 1990s, with the arrival and dispersal of the western flower thrips being one factor favoring this situation. During 2002 in South Australia, TSWV caused crop failures of up to 100% in glasshouse crops of capsicums with the Tsw gene for resistance to TSWV. These Tsw virulent strains were shown to cause severe systemic infection on Capscium chinense genotypes PI 152225 and PI 159236, which have the Tsw gene. Minimal differences were found between the nucleocapsid (N) gene amino acid sequences of the Tsw virulent and avirulent TSWV strains from Australia. Other biological and serological similarities were also shown between Tsw virulent and avirulent TSWV strains, including a hypersensitive reaction on TSWV resistant tomatoes with the Sw-5 gene. This is the first field record of Tsw virulent strains of TSWV in Australia. Capsicum chlorosis virus (CaCV) was first detected in Australia in 1999 (McMichael LA *et al*. 2002. *Australasian Plant Pathology* 31: 231–239). In the state of Queensland it has caused epidemics and symptoms, in capsicum crops, similar to those caused by TSWV. However, in most instances it infects up to 5–10% of capsicum plants in a crop. It is a distinct member of serogroup IV, with N gene nucleotide and amino acid sequence identities of <85% with other members of this group, and is most closely related to Watermelon silver mottle virus. CaCV infects all capsicum and tomato cultivars tested, including those with the TSWV resistance genes Tsw and Sw-5. However, resistance has been found in one C. chinense PI line from the USDA germplasm collection. Natural hosts are capsicum (pepper), tomato, groundnut and Hoya spp. CaCV is transmitted by Thrips palmi and Frankliniella schultzei. Transmission tests with WFT have been negative. CaCV affects all capsicum production areas in Queensland and recent records from other states (Western Australia and New South Wales) and Thailand, suggest a wide distribution in northern Australia and South East Asia.

# Research article: Views on the parameters of vector competency

**DOI:** 10.1673/031.007.2872

**Published:** 2007-05-04

**Authors:** JL Sherwood

**Affiliations:** Department of Plant Pathology, The University of Georgia, Athens, Georgia, USA sherwood@uga.edu

The number of thrips species that are known to transmit *Tomato spotted wilt virus* (TSWV) or other tospoviruses is limited, and the basis of competency of thrips to transmit tospoviruses has been investigated to any significance in only a few thrips-virus pathosystems. It has long been observed that only larval thrips that acquire tospoviruses or adults derived from such larvae are competent to transmit virus. Nonviruliferous adults do ingest virus particles when feeding upon virus infected plants, but such adult thrips have not been shown to transmit virus. Acquisition of virus by thrips has been shown to require the glycoprotein of the virus envelope (Sin S-H. 2005. *Proceedings of the National Academy of Sciences USA* 102:5168–5173), but if acquisition is a result of interaction with a ligand in the thrips midgut or a pH dependent process has not been fully elucidated. Upon entering the larval midgut, it has widely documented that virus replication ensues in the midgut tissues. This can be transient, with subsequent virus replication ensuing in the muscle fibers surrounding the midgut. Similar events have been noted when adult thrips and a non-vector species of thrips (see de Assis FM et al. 2004. *Phytopathology* 94:333–336, and references therein) have fed on virus-infected plants, but unlike when virus acquisition occurs in larval thrips of species that transmit the virus, virus is not subsequently found in the salivary glands. Several models have been proposed regarding how virus eventually becomes established in the salivary glands for subsequent transmission. The virus could transverse several membranes and move in the hemocoel to the salivary glands like other insect borne plant-infecting viruses that are persistently transmitted. A ligament-like structure that putatively forms a connection between the midgut and the salivary glands has been proposed as a tentative pathway for virus movement. Additionally, the proximity of the infected midgut organs to the salivary glands during thrips development may facilitate virus movement to the salivary glands. Each of these provides possibilities for understanding virus transmission, or lack thereof, by thrips. Additional insight on virus acquisition and vector competency can be obtained from virus isolates that form particles, but are not transmitted.

# Research article: Practical application of the ITS-RFLP method for rapid identification of thrips larvae

**DOI:** 10.1673/031.007.2873

**Published:** 2007-05-04

**Authors:** M Stuckey, J Funderburk

**Affiliations:** ^1^ North Florida Research and Education Centre, University of Florida, Quincy, Florida, USA. mlstuckey@mail.ifas.ufl.edu

Effective management and control of pest thrips is crucial to many agricultural projects, and accurate and rapid identification of adults and larvae is important in determining the early stages of infestations. This had previously been difficult, until the development of an interactive, computerized identification system (Moritz G *et al*. 2004. *Pest thrips of the world: an identification and information system using molecular and microscopical methods*), which combines morphological and molecular methods. The molecular component relies on restriction fragment length polymorphism (RFLP) of the internal transcribed spacer (ITS) regions of ribosomal DNA. We evaluated the utility and effectiveness of this ITS-RFLP method of identifying thrips for both a laboratory and a field based study.

# Research article: Ecological and physiological factors influencing transmission of *Tomato spotted wilt virus* (TSWV) by *Frankliniella fusca* and *F*. *occidentalis*

**DOI:** 10.1673/031.007.2874

**Published:** 2007-05-04

**Authors:** CF Stumpf, GG Kennedy

**Affiliations:** Department of Entomology, Box 7630, North Carolina State University, Raleigh, NC cfstumpf@ncsu.edu

*Tomato spotted wilt virus* (TSWV) replicates in its thrips vectors. Findings will be presented that demonstrate both direct effects and host plant-mediated effects of TSWV infection on thrips performance as measured by development time, survival, and adult size for *Frankliniella fasca*, but only host plant-mediated effects for *Frankliniella occidentalis*. The magnitude of these effects and their consequences on transmission rates are influenced to varying degrees by the main effects and interaction effects of temperature, host plant, and TSWV isolate. The potential implications of these findings for the epidemiology of TSWV will be discussed.

# Research article: Effect of organic and conventional production methods on thrips (Thysanoptera: Thripidae) species occurrence in field grown cut flowers

**DOI:** 10.1673/031.007.2875

**Published:** 2007-05-04

**Authors:** S Sutliff-Shipley, C Casey

**Affiliations:** Department of Entomology, Box 7613, North Carolina State University, Raleigh, NC 27695 USA. ssutlif@ncsu.edu

Field cut flower production in North Carolina is a growing industry as tobacco farmers move to flower production. Thrips are the most serious insect pest problem for these growers as they feed directly on flowers, which causes scarring and distortion, thereby reducing the crop's value. Several species of thrips also vector fatal tospoviruses such as impatiens necrotic spot virus (INSV) and tomato spotted wilt virus (TSWV). Little is known about thrips species occurrence in cut flower production. A preliminary survey of cut flowers in organic production indicates that the most predominant species of thrips is eastern flower thrips (*Franklinilla tritici*), which is not a tospovirus vector. However, it is unknown which species are predominant in conventional production and if farm location, soil type, and history also factor into thrips species distribution. The objective of this investigation is to identify the thrips species that occur in both organic and conventional field flower production in North Carolina. Seven farms (3 organic and 4 conventional) were sampled during the 2005 cut flower growing season. Thrips were sampled indirectly via yellow sticky cards as well as directly by biweekly sampling of 3 crop plant and 3 weed species. Knowledge of species distribution provided by this investigation will be a starting point for improved thrips management programs for both conventional and organic growers.

# Research article: Thrips and *Tospovirus* in Southern Australia with the Main Emphasis in the Sydney Basin

**DOI:** 10.1673/031.007.2876

**Published:** 2007-05-04

**Authors:** T Swaminathan, G Murdoch, A Clift

**Affiliations:** Agricultural Entomology Group, McMillan Building A05, Science Rd, University of Sydney, Sydney, New South Wales 2006, Australia Stha5139@mail.usyd.edu.au

There are three vector thrips and two *Tospoviruses* in southern Australia, which is defined as south of latitude 30°S. The vector thrips are onion thrips, *Thrips tabaci* Lindeman, tomato thrips, *Frankliniella schultzei* (Trybom) and western flower thrips (WFT) *F. occidentalis* (Pergande). The Tospoviruses are Tomato Spotted Wilt Virus (TSWV) and Iris Yellow Spot Virus (IYSV). There is a third Tospovirus, Capsicum Chlorosis Virus, which may be vectored by melon thrips, *T. palmi* Karny, but they both occur in Bundaberg and Childers, south east Queensland, well north of 30°S and will not be considered here. Main host plants are lettuce, potato, peppers, tomatoes and cut flowers such as chrysanthemums. All three vector species occur in the intensive horticultural production near Sydney, Melbourne, Adelaide and Perth. However, in the inland, irrigated production areas, including Crookwell, Hay and Jerilderie, the only vectors are *T. tabaci* and *F. schultzei*. WFT was found in Mildura earlier this century and has recently been found in the irrigated areas of Boort, northern Victoria in 2003. The main Tospovirus is TSWV, with IYSV present at on onion seed production areas around Narromine and Griffith. Please refer to the map for the location of these areas. TSWV is vectored by all three vectors, but IYSV is vectored only by *T. tabaci*. Contrary to popular belief, when infected, *F. schultzei* is as efficient vector as WFT, but because of its migratory habit, it is less likely to be infected. *T. tabaci* is intermediate- its is not as efficient vector, but as it moves readily from host to host, especially as weeds dry out during late spring (October/November) it does move the TSWV between both host plants and farms. WFT was first found in Australia in 1993, first in Perth, then Sydney, as two introductions. Its movement has been mainly by infested plant material, often via central markets. In the inland irrigation area, large, overnight flights of *F*. *schultzei* can introduce TSWV over a wide area at high levels of infection >80% infection of tomatoes if not sprayed. These flights involving infective thrips occur in about 20% of seasons; *T. tabaci* is often responsible for lower levels of TSWV, 1–25%, but at higher frequency. The major vector in Tasmania, in the Hobart and Launceston area, is *T. tabaci*, although there are some small, well contained infestations of WFT. In Sydney basin, the flight activity of the thrips population and the presence of TSWV were monitored fortnightly in three commercial horticulture farms. The seasonal dynamics of thrips, their movement pattern within and outside the farm, presence weed hosts for thrips and incidence of TSWV were discussed.

# Research article: Thrips-plant-interactions: Evolution of host selection, feeding and mating behavior

**DOI:** 10.1673/031.007.2877

**Published:** 2007-05-04

**Authors:** I Terry

**Affiliations:** University of Utah, Department of Biology, Salt Lake City, Utah 84112 USA. terry@biology.utah.edu

A review of thrips-plant interactions reveals that thrips occupy many of the feeding niches available to them on plants, and that in an evolutionary sense, thrips have been very opportunistic. From their early beginnings as likely fungal feeders in dtritus, thrips have diversified onto numerous plant groups as plants have evolved from non-seed bearing plants to the most advanced plant taxa. The major trends in thrips evolution and plant evolution will be reviewed in a phylogenetic context. The selection process by which thrips choose sites for feeding, mating and brood development is also very diverse, with some thrips species being highly specialized on specific hosts, to others that are archetypal generalists. A few case studies will be used as examples to demonstrate this diversity between thrips species specializing on a single host plant species or genus and those that feed, mate and breed on a wide array of plant species and families, and what is known about cues that these thrips use to select their host plants.

# Research article: *Macrozamia* cycad cone traits mediate thrips and weevil pollinator behavior

**DOI:** 10.1673/031.007.2878

**Published:** 2007-05-04

**Authors:** I Terry, C Moore, G Walter, C Hull, R Roemer

**Affiliations:** Department of Biology, University or Utah, 257 South 1400 East Street, Salt Lake City, UT 84112, USA. terry@biology.utah.edu

Because cycads are the basal clade of extant seed plants, and insects vector pollen between male and female cones, studies of cycad pollinators could reveal much about early pollination mutualisms. The cycad genus, *Macrozamia*, an Australian endemic, is unusual in having some species pollinated only by thrips in the genus *Cycadothrips*, whereas all other known cycad pollinators world-wide are beetles. Comparisons of several *Macrozamia* species pollinated only by *Tranes* sp. weevil with those pollinated by *Cycadothrips* sp. only reveal differences in their cone volatile components. *Tranes*-pollinated species release monoterpenes, primarily linalool and smaller amounts of beta-myrcene, whereas *Cycadothrips* —pollinated cycads lack linalool and release several monoterpenes dominated by very high levels of beta-myrcene. Diel periodicity of volatile emissions coincides with cone thermogenesis and insect movement between cones: daytime activity for *Cycadothrips*-pollinated species and evening activity for *Tranes*-pollinated ones. Results of electroantennograms (EAGs) and gaschromatography-electroantennographic detection (GC-EADs) show that insects respond physiologically to specific components within their host cone's volatile mix. In particular, *Cycadothrips chadwicki* respond to beta-myrcene and two beta-ocimene isomers, (E)- beta-ocimene and (allo)-beta-ocimene. *Tranes* sp. from *M. machinii* respond to both linalool and beta-myrcene. Olfactometer test results indicate that *Cycadothrips* are attracted by beta-ocimene isomers but are repelled by beta-myrcene. Weevils responded positively to linalool. The timing of specific component emissions may explain insect movements between cones. These tests suggest a possible plant species isolating mechanism that operates through the specific pollinator by affecting insect behavior.

# Research article: Plant volatiles and thrips; Are attractants arresting?

**DOI:** 10.1673/031.007.2879

**Published:** 2007-05-04

**Authors:** D Teulon, M Davidson, D James

**Affiliations:** New Zealand Institute for Crop & Food Research Limited, Private Bag 4704, Christchurch, New Zealand. davidsonm@crop.cri.nz

Anemotaxis is the principal behavior used by larger insects, such as lepidoptera, to locate mates, and presumably, suitable hosts. For smaller insects like thrips, orientation to a volatile compound using wind would be difficult because of their poor ability to fly. We propose that so called attractant volatile compounds potentially elicit an arresting response in flying thrips. Thrips' flight could be inhibited by such arrestants, so thrips do not fly in the presence of these compounds, or if they are in flight, these arrestants trigger a landing response. Arrestant activity has potentially been involved in thrips response to selected compounds in laboratory studies where fewer thrips flew in the presence of a volatile compound. Field trials were undertaken using black baited or unbaited water traps; or white unbaited water traps set at varying distances (0.5, 1, 2, 5, and 10 m) radiating out from a centrally baited trap. The volatile compound appears to elicit a landing response in flying thrips, because a higher number of thrips were recorded in baited black water traps. The central white water traps baited with a volatile compound had the highest numbers of thrips, with decreasing numbers recorded in unbaited traps within 2 m downwind of the central trap.

# Research article: Expression of *Tomato spotted wilt virus* RNA and protein in yeast from cDNA clones

**DOI:** 10.1673/031.007.2880

**Published:** 2007-05-04

**Authors:** AN Thompson, M Kainz, TL German

**Affiliations:** ^1^ Department of Entomology, University of Wisconsin-Madison, Madison, WI, USA.; ^2^ Department of Biology, Colgate University, Hamilton, NY, USA ant@entomology.wisc.edu

*Tomato spotted wilt virus* (TSWV) is a plant infecting virus in the family *Bunyaviridae* that replicates in its insect vector, *Frankliniella occidentalis*. Our long term goal is to produce infectious Tomato spotted wilt virus entirely from cloned components expressed from plasmids in yeast cells. We are using yeast cells because (1) they provide number of selectable markers to facilitate the delivery of all components required for the system from plasmids; (2) the system has been used successfully for positive sense RNA viruses and (3) the ability to use well established yeast genetic approaches to help understand the host genes involved in TSWV replication. We are using an approach similar to that used for animal viruses to establish the replicon system. We produced a product that was a faithful copy of the TSWV S RNA by reverse transcription PCR using primers that added a modified phage T7 promoter to generate the desired 5′ end. We then added a modified Hepatitis Delta Virus (HDV) ribozyme to generate the proper 3′ end and cloned the fragment into the yeast expression vector pRS426. Finally, we marked our construct by changing a single nucleotide in the NSs ORF to create a silent mutation that added a *PmlI* restriction site to the S sequences. This generated the plasmid TSWVREP, the basis of our replicon system. The TSWV N open reading frame was amplified from the plasmid pACT2-N, inserted behind the Gal1/Gal10 promoter, and cloned into yeast plasmid pLDB138 to create pTSWVN. The T7 RNA polymerase gene was amplified by PCR, modified to include the SV40 T antigen nuclear localization sequence, and inserted downstream from the ADH promoter to create pT7RNAP. Yeast cells (strain L2612) transformed with TSWVREP and pT7RNAP express the TSWV S RNA. Northern analysis verifies that S RNA extracted from viral RNPs isolated from infected plants and replicon RNA isolated from yeast co-migrate RT-PCR products using RNA from plant RNPs are not cleaved with *PmII* whereas RT-PCR products from the replicon S RNA extracted from yeast are digested with *PmII* as expected. Western analysis of yeast cells transformed with pTSWVN demonstrated that TSWV N protein is efficiently and stably expressed. Finally we have isolated an RNP-like structure from yeast transformed with all three plasmids that bands in a CsCl isopycnic gradient at the same density (1.32g/cc) as TSWV RNPs isolated from plants. Our data suggests that we are producing authentic, traceable, TSWV RNPs in yeast cells.

# Research article: Intra-specific genetic variation in *Thrips tabaci*

**DOI:** 10.1673/031.007.2881

**Published:** 2007-05-04

**Authors:** S Toda, T Murai, M Morishita

**Affiliations:** ^1^ Department of Grape and Persimmon Research, National Institute of Fruit Tree Science, Higashihiroshima, Hiroshima 729-2494, Japan; ^2^ Faculty of Agriculture, Utsunomiya University, Utsunomiya Tochigi 321-8505, Japan; ^3^ Laboratory of Persimmon and Peach, Fruit Tree Experiment Station, Wakayama Research Center of Agriculture, Forestry and Fisheries, Kokawa Wakayama 649-6531, Japan toda@affrc.go.jp

*Thrips tabaci* Lindeman is a widely-distributed pest insect that injures a wide range of crops. In Japan, it is known as a serious pest of *Allium*. However, this thrips has recently begun for the first time to infest other Japanese crops, such as persimmon and mandarin orange. It has been pointed out that there is genetic differentiation in *T. tabaci* (Brunner PC *et al*. 2004. *Heredity* 93: 364–370; Murai T and Toda S. 2002. In: Marullo R, Mound L, editors. *Thrips and Tospoviruses*: *Proceedings of the 7^th^ International Symposium on Thysanoptera*, pp. 377–378. CSIRO and Universita degli studi Mediterranea di Reggio Calabria). To gain an understanding of the genetic background underlying this phenomenon, we investigated the genetic variation of this species. We collected 6 overseas and 11 Japanese local populations. DNA was extracted from individual female adults and a part of mitochondrial cytochrome oxidase gene subunit I (COI) was amplified. The nucleotide sequences were analyzed by direct sequencing and compared among populations. As a result, four different haplotypes were detected in Japanese populations. Two haplotypes of persimmon-feeding populations differed from the majority haplotype of other Japanese populations and were identical to that detected in overseas populations. However, it is not clear whether or not the persimmon-feeding population is from overseas. In this study, we also analyzed the COI sequence of 10 male adults from two overseas and one Japanese population. Although their sequences showed considerable variation, they formed an independent clade with several females. We presume that this clade consists of arrhenotokous strains and the others are thelytokous strains.

# Research article: Efficacy testing of dDifferent IPM methods against *Thrips tabaci* Lindeman (Thysanoptera: Thripidae) on cabbage and onion

**DOI:** 10.1673/031.007.2882

**Published:** 2007-05-04

**Authors:** S Trdan, N Valic, D Znidarcic

**Affiliations:** University of Ljubljana, Biotechnical Faculty, Dept. of Agronomy, Jamnikarjeva 101, SI-1111 Ljubljana, Slovenia. stanislav.trdan@bf.uni-lj.si

In 2001–2002, the efficacy of light blue sticky boards for mass trapping of onion thrips in onion was investigated. In the drier year 2001, no differences in yield were determined between the treatments. In 2002, which was quite average regarding the weather conditions, the least damage on the leaves and the highest yield was in plants treated twice with deltamethrin. In the treatments with sticky boards the extent of damage on leaves was higher compared to the control treatment. It was concluded that adults from distant plants are attracted to the light blue color, but near the onion, the smell of plants prevails (Trdan S *et al*. 2005. *Zeitschrift für Pflanzenkrankheiten und Pflanzenschutz* 112: 173–180). In 2004, the efficacy of four intercrops to reduce the damage caused by thrips feeding was tested on the same host. In onion grown with buckwheat or lacy phacelia, the extent of damage on leaves was lower. In both treatments the yield of onion was significantly lower too, compared to onion grown with white clover or orchard grass, which were less efficient in deterring the pest from onion. Growing onion with buckwheat or lacy phacelia seems appropriate when the priority is a production of healthier food. When we aim to produce a higher yield, white clover or orchard grass are recommended as intercrops. The efficacy of deltamethrin was tested for control of onion thrips in early white cabbage too. In 2002, when the thrips population was numerous, no significant differences were found in the index of damage (considering the lower threshold), yield loss and net mass of heads between the plants sprayed once and twice. The threshold in both treatments was exceeded up to seventh leaf of head, the same as in the control plants. Considering the higher threshold, we recommend to remove four exterior leaves on cabbage heads sprayed once and twice. In the Slovenian growing region, as well as in other regions with similar climatic conditions, we recommend one application of deltamethrin in the first decade of June. In 2004, the efficacy of different mulches against the pest on early white cabbage was tested. The most damage was established on the exterior leaves of heads grown on black PE mulch and on unmulched soil. The mass of heads and net mass of heads were the highest in the PE mulch, while both parameters were the lowest in the soil covered with hay and barley straw. In the parallel experiment the soil was mulched with PE mulch one month after transplanting and after this time the organic mulches were put on the soil. No significant differences in both weight parameters and extent of damage on leaves were determined between PE mulch, hay and unmulched soil. The highest yield loss was found in cabbage plants, grown on artificial mulch and straw. The efficacy of IPM methods for thrips control in onion and cabbage is different and depends on many factors. The expedience of these methods in vegetable production depends on the pest population level, susceptibility of varieties (Trdan S *et al*. 2005. *African Entomology* 13: 85–95), agrotechniques and weather conditions. As the latter are more and more unforeseen, such is also the efficacy of IPM methods.

# Research article: Acetates in secretions of thrips

**DOI:** 10.1673/031.007.2883

**Published:** 2007-05-04

**Authors:** G Tschuch, P Lindemann, G Moritz

**Affiliations:** ^1^ Developmental Biology, Department of Biology,; ^2^ Phamaceutical Biology, Department of Pharmacy, Martin-Luther-University Halle-Wittenberg, D-06099 Halle, Germany. tschuch@zoologie.uni-halle.de

Secretions of Thysanoptera species consist of very different chemical constituents, such as aliphatic hydrocarbons, carboxylic acids, aliphatic esters, cyclic esters of hydroxyl carboxylic acids (lactones), pyranones, naphthoquinones, and terpenes. In this paper, only aliphatic esters of acetic acid, found in 30% of the investigated species, are the focus of attention. The aliphatic ester with longest chain known previously from Thysanoptera is octadecyl acetate from *Gynaikothrips uzeli*, only 2.2 % of secretion (Suzuki T *et al*. 1989. *Japanese Journal of Applied Entomology and Zoology* 24: 222–228). Hexadecyl acetate is reported from *Gynaikothrips ficorum* and *G. uzeli* where it is one of the main constituents, and in smaller amounts from *Leeuwenia (*=*Varshneyia) pasaniae* and from *Liothrips kuwanai* (Howard DF *et al*. 1987. *Phytophaga* 1: 163–170; Suzuki T *et al*. 1988. *Japanese Journal of Applied Entomology and Zoology* 23: 291–297). A small part of the *Liothrips kuwanai* secretion (1.2 %) is probably hexadecenyl acetate (double bond position unknown). The secretion of *L. pasaniae* contains mainly tetradecyl acetate (15.4 %), tridecane (51.3 %), and pentadecane (12.4 %). In contrast to these species of Tubulifera, the only species of Terebrantia from which chemicals have been identified so far, *Frankliniella occidentalis*, produce a mixture of dodecyl and decyl acetate in the anal droplets of second instar larvae, although only in traces in secretions by adults (Teerling CR *et al*. 1993. *Journal of Chemical Ecology* 19: 681–697.). The recently investigated defensive secretions of adult and larval *Suocerathrips linguis* (Phlaeothripidae, Thysanoptera) were found to contain a long chained acetate, (11Z)-11,19-eicosadienyl acetate, that was not previously known to occur naturally (Csuk R *et al*. 2004. *Tetrahedron* 60: 6001–6004; Tschuch G *et al*. 2005. *Journal of Chemical Ecology* 31: 1555–1565). This substance occurred together with octadecyl acetate and other long chained acetates. The eicosadienyl acetate repels ants and spreads on the surface of such potential predators. The mixture can provide a liquid long-lasting surface coating with a very low vapour pressure. In predators like ants and mites, using mainly the olfactory sense, coating the sensory organs would make the animals “blind”. They would not be able to find the prey again, and ants would have difficulty evaluating their own pheromones, such as trail pheromones to return to the nest and inform nest-mates about potential prey.

# Research article: Genetic diversity and evolution of *Tomato spotted wilt virus* (TSWV)

**DOI:** 10.1673/031.007.2884

**Published:** 2007-05-04

**Authors:** M Tsompana, I Carbone, J Abad, JW Moyer

**Affiliations:** ^1^ Department of Plant Pathology, University of North Carolina-Chapel Hill, Chapel Hill, NC; ^2^ Center for Integrated Fungal Research, Department of Plant Pathology, North Carolina State University, Raleigh, NC; ^3^ Department of Plant Pathology, North Carolina State University, Raleigh, NC mtsompa@ncsu.edu

*Tomato spotted wilt virus* (TSWV) is a leading plant viral pathogen (Moyer JW 1999. In: Webster R, Granoff, A, editors. *Encyclopedia of Virology*, 1803–7. Academic Press; Moyer JW. 2000. In: Hull R, editor, *Encyclopedia of Microbiology*, 592–597: Academic Press; Tsompana M *et al*. 2005. *Molecular Ecology* 14: 53–66) that infects more than 900 plant and 10 thrips species. TSWV has been shown to adapt to selection pressures by reassortment (Qiu WP *et al*. 1998. *Virology* 244: 186–194) and by accumulation of defective mutants in the population (Sin SH *et al*. 2005. *Proceedings of the National Academy of Sciences USA* 102: 5168–5173). The overall goal of this study was to elucidate the molecular evolution and population genetics of TSWV, at the species level and within individual isolates. Initially, to characterize the recent evolutionary history of the TSWV species we used consensus sequence data from genes encoding five viral proteins. Our analysis provided the first demonstration of population structuring and species-wide population expansions for TSWV, attributed to founder effects (Tsompana *et al*. 2005. *Molecular Ecology* 14: 53–66; Tsompana *et al*. 2003. *Phytopathology* 93. Supplement 6: 84). We also identified positive selection favoring divergence between *Tospovirus* species and purifying selection acting at the species level to preserve protein function. Subsequently, analyses of thirteen individual TSWV isolates (represented by 516 clones) revealed that natural TSWV isolates are highly heterogeneous viral populations that consist of one or two haplotypes with high frequency and an array of closely related rare haplotypes, some of which have deletions and/or stop codons. These viral populations exhibit a high transitional bias, related to the function of RdRp polymerase or dsRAD. Analysis based on tests of neutrality, gene genealogies and the coalescent uncovered a population history of genetic bottlenecks followed by population growth for all viral isolates, and a limited amount of recombination for three viral isolates. Migration was significantly restricted between viral populations. Overall, our findings improve our knowledge on the important factors that govern the evolution of plant RNA viruses.

# Research article: Analysis of *Tomato spotted wilt virus* (TSWV) transmission by *Thrips setosus*

**DOI:** 10.1673/031.007.2885

**Published:** 2007-05-04

**Authors:** S Tsuda

**Affiliations:** National Agricultural Research Center, Tsukuba, Ibaraki 305-8666, Japan. shinyat@affrc.go.jp

Tospoviruses are currently known to be transmitted by at least ten species of thrips. *Frankliniella occidentalis* (Pergande), the western flower thrips, having a broad host range is the most common tospovirus vector insect. As documented for *F. occidentalis*, when *Thrips setosus* (Moulton), the light-brown soybean thrips, which is an endemic species in Japan, acquires *Tomato spotted wilt virus* as a first instar larvae, the virus enters the midgut epithelial cells, replicates, and moves to salivary glands during pupation, then the emerged adult is able to transmit the virus. The previous cellular investigations carried out only in *F. occidentalis* if acquisition access feeding (AAF) was first given as an adult suggested that infectivity was prevented because TSWV was unable to enter the midgut of adult thrips. In adult *T. setosus* given an AAF as an adult, however, the virus was found to enter and replicate in the midgut, but not in the salivary glands meaning that could not transmit the virus. Electron microscopic observations of adult *T. setosus* showed that no infection occurred in cells beyond the midgut epithelia, suggesting that the virus infection is limited in the epithelial cells. In contrast, when larval *T. setosus* was first given an AAF, the virus was found to the midgut and the visceral muscle tissue lining the midgut as that of larval *F. occidentalis* infected. Our findings suggest that the barrier to infectivity in adults *T. setosus* differs from that shown for adults *F. occidentalis*.

# Research article: Host plant resistance to TSWV: current status and historical perspective

**DOI:** 10.1673/031.007.2886

**Published:** 2007-05-04

**Authors:** Turina Massimo, Ciuffo Marina, Margaria Paolo

**Affiliations:** Istituto di Virologia Vegetale, CNR-Torino. Strada delle cacce 73, 10135 Torino-Italy m.turina@ivv.cnr.it

Due to the difficulty of controlling the thrip vector, with either chemical or biological control methods, host plant resistance to TSWV is the only economically feasible approach to controlling *Tomato spotted wilt virus* (*Tospovirus*, *Bunyaviridae*). Through traditional breeding programs a variety of resistance genes from different plant species have been introgressed into the vegetable crops most heavily damaged by this virus. We will briefly outline the history of the discovery and the characterization of the two most popular resistance genes: *Sw5* in tomato, and *Tsw* in pepper. Over the years, reports of the presence of resistance breaking strains of TSWV and the resulting disease epidemics have stimulated the search for new resistance genes for both tomato and pepper crops. We will provide an overview of the importance of such epidemic episodes, assess the probability of finding and introgressing new resistance genes, and discuss potential outcomes of such efforts. Additionally, in the case of these resistance breaking strains of TSWV infecting both resistant pepper and tomato plants, the avirulence-resistance gene interaction at the molecular level is of particular interest; we will assess past and recent acquisitions of such an interaction. Most recently, pathogen derived resistance against TSWV has also been experimentally employed: we will report the outcome of the different approaches used for this defense strategy and the possibilities of its open field implementation. The emergence of new *Tospoviruses* and potential host resistance to such viruses will also be discussed.strategy and the possibilities of its open field implementation. The emergence of new *Tospoviruses* and potential host resistance to such viruses will also be discussed.

# Research article: Thrips Vectors and Tospoviruses: On the Edge of Adaptation

**DOI:** 10.1673/031.007.2887

**Published:** 2007-05-04

**Authors:** D Ullman

**Affiliations:** Department of Entomology, University of California, One Shields Avenue, Davis. CA 05616-8584 USA. deullman@ucdavis.edu

The coevolution of insect vectors and the viruses they transmit represents one of the most fascinating systems in which evolution and its consequences can be studied in observable time spans (reviewed in Whitfield AE *et al*. 2005. *Annual Review of Phytopathology* 43: 17.1–17.31). Evolutionary processes in viruses are at the root of their of their pathogenic potential and certainly this is true of viruses in the genus *Tospovirus*. Although less is known about the thrips genome, their diversity and rapid adaptation to environmental challenges (e.g. pesticide resistance) suggests evolutionary processes also fuel their success and emergence as pests. Testimony to the likely importance of thrips-tospovirus coevolution can be found in the emergence of at least 13 new tospovirus species and at least three new thrips vector species over the past 15 years. Although tospoviruses can be mechanically transmitted under experimental conditions, tospovirus dispersal and survival in nature depends on passage to plants by thrips vectors. The dispersal and survival of TSWV depends upon: a) coexistence of virus and vector populations under conditions of genetic and physiological compatibility; and, b) environmental and plant-host interactions with the virus and the insect allowing every phase of the infection cycle. Thus, coevolution between thrips and tospoviruses must greatly influence the observed variability between virus isolates, epidemics, and even the emergence of new tospoviruses. Tremendous genetic variability in tospovirus populations is provided by the high error rate inherent in RNA replication by the viral RdRp and the reassortment of genomic segments between different virus isolates in planta (Ullman D *et al*. 2005. *Proceedings of the National Academy of Science USA* 102:4931–4932). Sin *et al*. (2005, *Proceedings of the National Academy of Science USA* 102:5168–5173) showed that the genetic determinants of insect transmission of TSWV lie on the viral M RNA. In mixed infections of a thrips transmissible and a thrips non-transmissible isolate reassortment occurred and a specific mutation on the M RNA was shown to eliminate insect transmissibility. Little is known about the thrips genome, but all of the vector species are characterized by striking morphological diversity, which suggests genetically variable populations. Tospovirus and thrips vector populations certainly meet the basic requirements for rapid coevolution. It seems likely coevolution between thrips and tospoviruses, the potential for TSWV reassortment, and the role of M RNA in determining transmissibility all play critical roles in the emergence of new vector-virus relationships.

# Research article: The curious incident of the thrips and the *Tospovirus*

**DOI:** 10.1673/031.007.2888

**Published:** 2007-05-04

**Authors:** D Ullman

**Affiliations:** Department of Entomology, University of California, One Shields Avenue, Davis, CA 95616-8584 USA. deullman@ucdavis.edu

Insect vectors play a key role in dissemination of viruses causing important diseases of humans, animal and plants. Specific understanding of insect-virus interactions leading to successful transmission is a central problem in vector biology and critical to developing novel control strategies (Ullman DE, et al. 2005. *Proceedings of the National Academy of Science USA* 102: 4931–4932). *Tomato spotted wilt virus* (TSWV) is the type member of the genus *Tospovirus* in which at least 15 virus species are proposed. The *Tospoviruses* are transmitted between plants by at least 10 species of thrips (Thripidae, Thysanoptera) and are the only plant-infecting members in the family *Bunyaviridae*. The cellular and molecular determinants underlying the thrips — tospovirus relationship and vector competence are biological mysteries fueling intense research by entomologists and virologists worldwide. Certainly, the complex nature of the interplay between thrips, tospoviruses and their shared plant hosts make them ideal model systems for elucidating processes of virus infection in disparate hosts that can be extended to solutions for tospovirus epidemics in crops and to controlling viruses of importance to human health. The discoveries that have advanced our understanding of the interface between thrips and the tospoviruses they transmit have been driven by an understanding of thrips internal anatomy and physiology and the genome strategies and biology of the tospoviruses. The finding that tospoviruses multiplied in their thrips vectors opened rich and exciting avenues of exploration, including understanding biological and molecular interactions underlying TSWV pathogenesis in plant and insect hosts and the role these processes play in virus evolution. Discoveries during the last decade show that insect inoculation of tospoviruses into a plant host cannot occur without viral passage across at least three insect organs (the midgut, visceral muscle cells and salivary glands) that include six membrane barriers (Whitfield AE et al. 2005. *Annual Review of Phytopathology* 43: 17.1–17.31). Attention on thrips internal anatomy, development and thrips-tospovirus interactions have revealed much about the pathway of the virus in the insect, the membrane barriers to virus passage and the importance of ontogeny to virus acquisition and vector competence. The hypothesis that tospovirus glycoproteins are essential determinants of thrips acquisition has been advanced significantly through two important breakthroughs: a) Whitfield *et al*. 2005 (*Virus Research* 110:183–86) developed a system to express the GPs, study their structure and function and experimentally manipulate their interactions with the insects; and, b) Sin *et al*. 2005. (*Proceedings of the National Academy of Science USA* 102:5168–5173) refined use of viral reassortants to study the impact of specific virus mutations on thrips acquisition. Stumpf CF and Kennedy GG. 2005. (*Entomologia Experimental et Applicata* 114:215–25) used this same system of reassortants to advance our knowledge of the interactions between virus genetic diversity and ecological and physiological factors impacting thrips transmission of tospoviruses. Clearly, we have entered an era in which understanding of TSWV-thrips vector interactions can advance as never before. Translation of these fundamental advances into creative new control strategies will be a significant, but exciting challenge in the years to come.

# Research article: Identification of *Thrips palmi*: Morphology Compared with Real-Time PCR

**DOI:** 10.1673/031.007.2889

**Published:** 2007-05-04

**Authors:** G Vierbergen, HE van den Beld, BTLH van de Vossenberg, LFF Kox

**Affiliations:** Plant Protection Service, P.O. Box 9102, 6700 HC Wageningen, The Netherlands. g.vierbergen@minlnv.nl

In juvenile stages the EU IA1 quarantine organism *Thrips palmi* is increasingly more intercepted at port of entry in Europe. Because of the difficulties with identification of these stages notifications result in reference in higher taxonomic levels. In The Netherlands a real-time PCR assay is developed, which enables the identification to species levels of every stage. To test the sensitivity inserted eggs in leaf tissue were detected by amplification of the cut out plant tissue. Real-time PCR assay resulted in the detection of *T. palmi* eggs in 19 of 56 cut out tissues from ‘naturally’ infested imported eggplant-fruit. The assay does not have any influence of environmental factors affecting development of the thrips, no genetic variation is established (Knox LFF *et al*. 2005. *OEPP/EPPO Bulletin* 35:141–148). The method can be standardized and needs less extensive experience than morphological identification. Disadvantages are the longer analysis time, the strictly presented results, the sub-specificity and the higher costs. Quarantine laboratories asked for identification of *T. palmi* have to weigh the advantages and disadvantages of employing PCR or morphological expertise or both.

# Research article: Spring precipitation affects *Frankliniella fusca* (Thysanoptera: Thripidae) population growth and *Tomato spotted wilt virus* spread within patches of the winter annual weed *Stellaria media*

**DOI:** 10.1673/031.007.2890

**Published:** 2007-05-04

**Authors:** SC Voss, GG Kennedy

**Affiliations:** North Carolina State University, Research Annex West, Raleigh, North Carolina scvoss@ncsu.edu

In North Carolina, winter annual weeds, including common chickweed, *Stellaria media*, are the primary source of TSWV inoculum that is spread to susceptible crops in spring by *Frankliniella fusca* Hinds. To test the hypothesis that rainfall influences population growth of *F. fusca* and spread of TSWV among winter annual weeds, precipitation levels and timing were manipulated to mimic 2002, a year during which *F. fusca* populations and TSWV incidence in crops reached exceptionally high levels, and 2003 when *F. fusca* populations and TSWV incidence were unusually low. Rainfall levels were manipulated weekly using a combination of rain shields and overhead sprinklers. Seven experimental treatments were established over identical patches of common chickweed containing a single source of TSWV and infested with *F. fusca*. The treatments included: low level of precipitation mimicking rainfall patterns of 2002; high level of precipitation mimicking 2003 rainfall; and ambient rainfall. Other treatments included 2003 patterns of rainfall during only February, March, April, or May, with 2002 rainfall patterns during other months. Regression analysis revealed a strong and significant effect of both rainfall amount and number of days with rain on the increase in *F. fusca* populations and spread of TSWV within chickweed patches. Knowledge of this relationship may be useful for predicting when susceptible crops are at highest risk of TSWV infection.

# Research article: Circadian Sex

**DOI:** 10.1673/031.007.2891

**Published:** 2007-05-04

**Authors:** KW Webster, P P Cooper, LA Mound

**Affiliations:** CSIRO Entomology, Canberra, Australia. Laurence.Mound@csiro.au

Kelly's citrus thrips is a major pest of citrus fruit in southern Australia and several Mediterranean countries. Adults are sexually dimorphic and have been demonstrated to have a peak period of sexual activity at 17.00h. Males produce one or more chemical attractants that induce male aggregation and also attract females to these aggregations for mating.

# Research article: Resistance to the thrips vectors of tomato spotted wilt virus (TSWV) in potato

**DOI:** 10.1673/031.007.2892

**Published:** 2007-05-04

**Authors:** GC Westmore, GR Allen, NW Davies, CR Wilson

**Affiliations:** ^1^ Tasmanian Institute of Agricultural Research, University of Tasmania, Private Bag 98, Hobart, Tasmania 7001, Australia.; ^2^ Central Science Laboratory, University of Tasmania, Private Bag 74, Hobart, Tasmania 7001, Australia. Calum.Wilson@dpiw.tas.gov.au

In potato, infections with Tomato spotted wilt tospovirus (TSWV) are sporadic, but losses are considerable (Wilson 2001). Potato cropping regions of south-eastern Australia are particularly prone to TSWV epidemics (Wilson CR. 2001. *Plant Pathology* 50: 402 — 410). Typically TSWV infections in potato are clustered along crop borders, with no aggregation within the crop and infections are associated with summer thrips flights. This suggests a major role of external virus inoculum, and limited internal secondary spread of infection. However, in some varieties, TSWV spread from infected potato has been noted. Whilst four of the known TSWV thrips vector species are found in Australia (Mound LA. 1996. *Acta Horticulturae* 431: 298 — 309), only two (*Thrips tabaci* and *Frankliniella schultzei*) are associated with potato in Southern Australia, and in Tasmania only *T. tabaci* (Wilson 2001). There are no current effective methods available for controlling TSWV in potato. Host resistance provides a promising long-term strategy for TSWV management (Cho JJ *et al*. 1989. *Plant Disease* 73: 375–83). Most breeding programs focus on virus resistance (eg. Black LL *et al*. 1991. *Plant Disease* 75: 863), however resistance to the vector can provide valuable disease control (Jones AT. 1987. *Annuals of Applied Biology* 111: 745–772). Differences in the perception and performance of *T. tabaci* on potatoes has been previously described (Jericho C. 2005. Epidemiology and the development of risk assessment models for the management of tomato spotted wilt virus (TSWV) in potatoes. PhD Thesis, University of Tasmania). Field performance of some potato varieties highly susceptible to TSWV infection when mechanically challenged, has seen few plants succumb to infection within field trials. Other field trials have shown differences in number of onion thrips and feeding damage across potato cultivars, suggesting resistance to the thrips vector was most likely. Resistance may be in the form of attraction/deterrence and/or resistance to thrips reproduction. A range of plant characteristics are known to alter thrips landing, feeding and reproductive behaviour (eg. Brodbeck BV et al. 2001. *Entomologia Experimentalis et Applicata* 99:165–72; Koschier EH et al. 2002. In: *Marullo R*, *Mound L*, *Editors*. *Thrips and tospoviruses*. *Proceedings of -the seventh International Symposium on Thysanoptera*, 185–187). Subtle volatile chemical differences in potato cultivars have been identified, particularly in the level of monoterpenes and sesquiterpenes. Paired colour preference trials have shown onion thrips differentiate between subtle colour differences, which may extend to potato cultivars ranging from light- to dark-green foliage, and light-green to dark-purple stems. Virus transmission trials have shown varying levels of vector competence in onion thrips populations sourced from different locations and from different hosts. Our efforts are now focused on a broad approach investigating colour preference, chemical deterrence, and factors underpinning vector competence.

# Research article: The role of *Tomato spotted wilt virus* (TSWV) glycoproteins in virus acquisition by thrips

**DOI:** 10.1673/031.007.2893

**Published:** 2007-05-04

**Authors:** AE Whitfield

**Affiliations:** Department of Entomology, University of Wisconsin, Madison WI 53706. aew@plantpath.wisc.edu

Viruses in the family *Bunyaviridae* encode envelope glycoproteins, GN and GC, which play essential roles in virus entry into host cells. One of the primary objectives of my research is to characterize the individual roles of *Tomato spotted wilt virus* (TSWV) GN and GC during early infection events of the thrips midgut. To this end, I expressed and purified a soluble, recombinant form of the GN protein (GN-S) to directly test the hypothesis that GN plays a role in TSWV acquisition by thrips. GN-S bound larval thrips midguts without the assistance of other viral proteins when fed to thrips in an *in vivo* binding assay ((Whitfield AE et al. 2004. *Journal of Virology* 78: 13197–13206). In addition, GN-S reduced larval acquisition of TSWV as revealed by immunolabeling of larval midguts (Fig. 1). To test the hypothesis that GN-S inhibits virus transmission by thrips, insects were given an acquisition access period (AAP) on TSWV and GN-S or TSWV alone and then tested for their ability to transmit TSWV to leaf discs. Fifty-six percent of thrips given an AAP on TSWV transmitted virus; however, only 6% of thrips given an AAP on the combination treatment of TSWV and GN-S transmitted virus. This is the first description of transmission inhibition by a glycoprotein of a bunyavirus. Vertebrate-infecting members of the Bunyaviridae enter host cells by pH-dependent endocytosis, and during this process, the glycoproteins are exposed to conditions of acidic pH within endocytic vesicles causing the GC protein to change conformation. To gain further insight into the role of the TSWV glycoproteins in virus acquisition, virions were subjected to varying pH conditions. TSWV GC was cleaved at acidic pH indicating that it undergoes a pH-dependent conformational change consistent with it serving as a fusion protein during pH-dependent endocytosis (Whitfield AE et al. 2005. *Virus Research* no: 183–186) GC also contains hydrophobic domains that may serve as fusion peptides during virus- and host-membrane fusion. Based on these findings, I hypothesize that TSWV enters thrips midgut cells in a pH-dependent manner and that GN serves as a viral attachment protein while GC functions as a fusion protein.

# Research article: Broad-spectrum resistance to distinct tospoviruses in transgenic tobacco carrying the conserved region of the L protein of *Watermelon silver mottle virus*

**DOI:** 10.1673/031.007.2894

**Published:** 2007-05-04

**Authors:** S-D Yeh, T-C Chen, J-C Peng, F-L Liu, Y-L Wu

**Affiliations:** Department of Plant Pathology, National Chung Hsing University, Taichung, 40227, Taiwan. sdyeh@nchu.edu.tw

Comparison of the deduced L proteins among tospoviruses revealed that the region containing reported motifs of RNA-dependent RNA polymerase (RdRp) is conserved. Accordingly, the DNA fragment corresponding to the conserved region (nts 3975–4928) of the viral complementary strand of L RNA of *Watermelon silver mottle virus* (WSMoV), the type member of the WSMoV serogroup, was cloned and modified as a translatable construct WLm in an *Agrobacterium* binary vector. In addition, duplicated stop codons or stop codons plus frame shift were added at the upstream of the conserved motifs region to generate untranslatable constructs WLmt and WLmts, respectively. Antisense and inverted repeat of the conserved region were also constructed and denoted WLmAs and WLmds, respectively. Transgenic lines of *Nicotiana benthamiana* carrying individual constructs were evaluated against tospoviruses under greenhouse conditions. In addition to WSMoV, other distinct tospoviruses including *Tomato spotted wilt virus* (TSWV), *Groundnut ringspot virus* (GRSV), *Impatiens necrotic spot virus* (INSV), and *Peanut chlorotic fan-spot virus* (PCFV) were used for assay. WLm, WLmt, WLmts, WLmAs, and WLmds transgenic lines showed various degrees of resistance against WSMoV, with 48.3% (14 out of 29 lines assayed), 55% (11/20), 60% (9/15), 66.7% (20/30), and 74.1% (20/27) lines, respectively. Among the lines resistant to WSMoV, there were 5 WLm lines, 4 WLmt lines, 1 WLmts line, 9 WLmAs lines, and 14 WLmds lines were resistance to TSWV; 1 WLmt line, 2 WLmAs lines, and 5 WLmds lines were resistant to GRSV; 1 WLmt line, 2 WLmAs lines, and 3 WLmds lines were resistant to INSV; and 1 WLmt line, 3 WLmAs lines, and 4 WLmds lines were resistant to PCFV. Moreover, 1 WLmt line was highly resistant to all five tospoviruses. Our results indicated that transgenic tobacco lines carrying the RdRp conserved region provide broad-spectrum resistances to different tospoviruses. This approach is being applied for economic crops, such as melon and tomato, for the control of different tospoviruses.

# Research article: Characterization of Calla lily chlorotic spot virus as a new tospovirus species

**DOI:** 10.1673/031.007.2895

**Published:** 2007-05-04

**Authors:** S-D Yeh, T-C Chen, Y-H Lin, H-T Hsu, C-C Chen

**Affiliations:** ^1^ Department of Plant Pathology, National Chung Hsing University, Taichung, 40227, Taiwan.; ^2^ US Department of Agriculture, Agricultural Research Service, Beltsville Agricultural Research Center, Beltsville, MD20705, USA; ^3^ Taichung District Agricultural Improvement Station, Changhua, 515, Taiwan sdyeh@nchu.edu.tw

A new tospovirus designated Calla lily chlorotic spot virus (CCSV) that caused chlorotic spots ranging from light green to yellow on the middle leaves of the affected calla lilies was isolated from central Taiwan. Virions of 75 to 105 nm in diameter were found present in crude extracts and ultra thin sections of diseased leaves by electron microscopy, and the transmission by *Thrips palmi* was evidenced. Polyclonal and monoclonal antibodies (MAbs) to the nucleocapsid protein (NP) of CCSV were produced. Weak cross-reaction was observed when the rabbit antiserum to the CCSV or *Watermelon silver mottle virus* (WSMoV) NP was used to react with the heterologous antigen in indirect enzyme-linked immunosorbent assay (ELISA) and immunoblotting. On the contrary, MAbs produced to CCSV NP or WSMoV NP reacted specifically with the homologous antigen but not with the heterologous antigen in both ELISA and immunoblot analyses. Results of reverse transcription-polymerase chain reaction (RT-PCR) showed that the conserved regions of the L genes of tospoviruses are present in CCSV, but primers specific to the N gene of WSMoV failed to amplify DNA fragments from total RNA extracts of CCSV-infected plants. The CCSV S RNA was determined to be 3,172 nts in length, with an inverted repeat at 5′ and 3′ ends and two open reading frames encoding the NP and a non-structural (NSs) protein in an ambisense arrangement. A typical 3′-terminal sequence (5′-AUUGCUCU-3′) shared by all members of the genus *Tospovirus* was also noticed. Sequence analysis indicated that the CCSV NP and NSs protein share low amino acid identities, 20.1 to 65.1% and 19.9 to 66.1%, respectively, with those of reported tospoviruses. Phylogenetic dendrogram analysis indicated that CCSV is a distinct member in the genus *Tospovirus*. We conclude that CCSV is a new tospovirus species belonging to the WSMoV serogroup.

# Research article: Production of serogroup-specific antibodies using the plant viral vector expressed non-structural NSs protein of *watermelon silver mottle virus*

**DOI:** 10.1673/031.007.2896

**Published:** 2007-05-04

**Authors:** S-D Yeh, T-C Chen, C-W Huang, Y-W Kuo, F-L Liu, C-H Yuan, H-T Hsu

**Affiliations:** ^1^ Department of Plant Pathology, National Chung Hsing University, Taichung, 40227, Taiwan; ^2^ Floral and Nursery Plants Research Unit, U. S. Department of Agriculture, Agricultural Research Service, Beltsville, Maryland 20705, U. S. A. sdyeh@nchu.edu.tw

Using the *Zucchini yellow mosaic virus* (ZYMV) vector, the S RNA encoded nonstructural (NSs) protein of *Watermelon silver mottle virus* (WSMoV) was successfully expressed in squash. The expressed NSs protein with a histidine tag was first isolated by the Ni2+-NTA affinity column, and further purified by sodium dodecyl sulfate-polyacrylamide gel electrophoresis (SDS-PAGE) for production of a rabbit antiserum and mouse monoclonal antibodies (MAbs). The rabbit antiserum specifically reacted with the crude antigen in the extract of WSMoV-infected *Nicotiana benthamiana*, and cross reacted with that of the high temperature-recovered gloxinia isolate (HT-1) of Capsicum chlorosis virus (CaCV). It is interesting to note that the ascitic fluids, produced from 3 stabilized hybridoma lines 231E6D12, 238C2G10 or 239F1C11, strongly reacted with extracts from plant tissues infected with WSMoV, CCSV or CaCV, all belonging to the WSMoV serogroup. Various deletions of the NSs open reading frame (ORF) were expressed by the ZYMV vector for epitope mapping. The results indicated that all three MAbs target at the amino acids 89–125 of the expressed WSMoV NSs protein. The serological analysis coupled with the sequence alignment revealed that the MAbs-targeted region of the NSs protein is highly conserved among four available sequences of WSMoV, CaCV, CCSV, and *Peanut bud necrosis virus* (PBNV) in the WSMoV serogroup. Therefore we conclude that the produced NSs-MAbs react with the common epitope of the NSs proteins and are useful for the detection of different tospovirus members in the WSMoV serogroup.

# Research article: Apple pollen as a supplemental food for the western flower thrips, *Frankliniella occidentalis*: Response of individuals and populations

**DOI:** 10.1673/031.007.2897

**Published:** 2007-05-04

**Authors:** J Zhi, GK Fitch, DC Margolies, JR Nechols

**Affiliations:** ^1^ Department of Plant Protection, Agricultural College, Guizhou University, Guiyang 550025, China; ^2^ Department of Biology, Avila University, 11901 Wornall, Kansas City MO 64145; ^3^ Department of Entomology, Kansas State University, 123 West Waters Hall, Manhattan KS 66056. dmargoli@oznet.ksu.edu, jnechols@oznet.ksu.edu

Experiments were performed to investigate the influence of apple pollen, plant diet, and relative humidity on individual life history traits and on population growth of the western flower thrips, *Frankliniella occidentalis* (Pergande) (Thysanoptera: Thripidae). In experiments with individual thrips, availability of bean pods plus a mixture of apple pollen and lycopodium shortened larval development time, and hence time from egg to adult, compared to bean pods alone. Similarly, total number of eggs laid and mean number of eggs laid per female per day were greater in the presence of pollen plus bean pods than on bean pods alone. Diet did not affect survival or length of the preoviposition and oviposition periods. A diet including bean pods plus pollen shortened the generation time and population doubling time, and increased net reproductive rate and the intrinsic rate of increase, compared to bean pods alone. In experiments on populations, growth was measured with different amounts of pollen on bean pods under two relative humidities, ca. 38 and 66%, and on two bean substrates, pods or leaves, at 70% RH. The presence or amount of pollen had no effect on population growth regardless of the other variables. Greater population growth occurred under the higher relative humidity regardless of substrate. Population growth was greater on bean pods than on bean leaves. Despite reports on the nutritional benefit of pollen for *F. occidentalis*, this benefit may be affected by pollen type, host plant, and other factors. In situations in which pollen has little influence on the growth of *F*. *occidentalis* populations, pollen that benefits a thrips predator could be used in an integrated management plan for controlling thrips.
